# Mitochondrial diseases: expanding the diagnosis in the era of genetic testing

**DOI:** 10.20517/jtgg.2020.40

**Published:** 2020-09-29

**Authors:** Russell P. Saneto

**Affiliations:** 1 Center for Integrative Brain Research, Neuroscience Institute, Seattle, WA 98101, USA.; 2 Department of Neurology/Division of Pediatric Neurology, Seattle Children’s Hospital/University of Washington, Seattle, WA 98105, USA.

**Keywords:** Mitochondria, oxidative phosphorylation, electron transport chain, genetic pathological variants, phenotype, genotype

## Abstract

Mitochondrial diseases are clinically and genetically heterogeneous. These diseases were initially described a little over three decades ago. Limited diagnostic tools created disease descriptions based on clinical, biochemical analytes, neuroimaging, and muscle biopsy findings. This diagnostic mechanism continued to evolve detection of inherited oxidative phosphorylation disorders and expanded discovery of mitochondrial physiology over the next two decades. Limited genetic testing hampered the definitive diagnostic identification and breadth of diseases. Over the last decade, the development and incorporation of massive parallel sequencing has identified approximately 300 genes involved in mitochondrial disease. Gene testing has enlarged our understanding of how genetic defects lead to cellular dysfunction and disease. These findings have expanded the understanding of how mechanisms of mitochondrial physiology can induce dysfunction and disease, but the complete collection of disease-causing gene variants remains incomplete. This article reviews the developments in disease gene discovery and the incorporation of gene findings with mitochondrial physiology. This understanding is critical to the development of targeted therapies.

## INTRODUCTION

Mitochondria are specialized organelles found in all nucleated mammalian cells. Described initially in 1890 and named in 1894, these organelles were shown to house the machinery for oxidative phosphorylation in the late 1940s^[1]^. The advent of clinical mitochondrial disease in medicine did not occur until decades later, when a Swedish woman was described with severe hypermetabolism, yet euthyroid^[2]^. To this day, the mechanism of her disorder remains a mystery. The seeds of mitochondrial molecular disease were planted with the description of mitochondria possessing their own DNA^[3,4]^. Within several decades, the entire mitochondrial genome was sequenced^[5]^. The genetic era of mitochondrial disease was ushered in 1988, when pathological variants in two diseases were described. Nine patients with myopathy were proven to have a single large deletion in mitochondrial DNA (mtDNA) and 33 patients having optic neuropathy with a pathological variant in a single subunit of Complex I, ND4, in the electron transport chain (ETC)^[6,7]^. The description of a pathological variant in a nuclear-encoded gene followed within the next year. A variant in *pyruvate dehydrogenase E1* was described in a boy who developed lactic acidosis with exercise^[8]^. In 1995, the first pathological variants in an ETC nuclear-encoded gene *succinate dehydrogenase subunit A* (*SDHA*) in Complex II was described in two sisters with Leigh syndrome^[9]^. The identification of mitochondrial diseases has rapidly expanded with gene discovery technological improvements over the last two decades. Disorders of mitochondrial function are considered the most common group of inborn errors of metabolism, with an estimated minimum disease prevalence in adults of ~12.5 per 100,000 and ~4.7 per 100,000 in children^[10,11]^. The difficulty in making a confirmed genetic diagnosis makes prevalence estimates low^[12]^.

## DIAGNOSIS OF MITOCHONDRIAL DISEASES: HISTORICAL OVERVIEW

The field of mitochondrial medicine has grown through the improvements in biochemical analysis, neuroimaging, gene discovery, genetic manipulation/engineering, and cell biology. Until the early 2000s, the first mitochondrial diseases were based on clinical-biochemical-pathological correlations. Diagnosis was based on the combination of analyte (e.g., abnormal plasma amino acid and organic acid patterns, serum and/or lactic acid levels), nuclear magnetic resonance imaging (MRI) findings, and muscle pathological findings (e.g., ragged red and blue fibers, paracrystalline structures, and abnormal cristae). Utilization of these testing methods began mitochondrial disease nosology with clinical-pathological correlations defining multiple mitochondrial syndromes, for example chronic progressive external ophthalmoplegia (CPEO) and, when accompanied by myopathy, CPEO plus. Leigh syndrome, first described by Denis Leigh, was defined initially by pathological findings and later by MRI changes, together with the clinical symptoms of a rapidly progressive stepwise neurodegenerative process^[13]^. This strategy led to the definition of the initial classic mitochondrial syndromes, which have stood the test of time [Table 1].

In the early years of diagnostic investigation, the tools used to validate disease mostly involved muscle dysfunction combined with biochemical and clinical findings-so much so that, during the decades preceding the 2000s, primary detection and confirmation of disease was based on findings from muscle biopsies together with clinical, biochemical, and imaging findings. Histochemical and electron microscopy analysis of muscle, together with the enzymatic activity of ETC complexes, also known as the respiratory chain, were folded into the diagnostic rubric of clinical, biochemical, and neuroimaging findings^[14]^. Clinicians used these data to further classify patients who did not fit one of the syndromes, into ETC abnormalities as Complex I, II, III, IV, or V disease^[15,16]^. Together with the near constant involvement of the central nervous system, this led to the term “mitochondrial encephalomyopathy” to describe mitochondrial diseases^[17]^.

Analysis and confirmation of disease during this time required clinical acumen guided by laboratory analysis. Analyte testing alone is not sufficiently sensitive or specific to absolutely confirm the diagnosis. In fact, to date, there is no single biomarker that confirms the diagnosis of mitochondrial disease. Muscle biopsy and analysis was part of the standard evaluation of patients being worked up for possible mitochondrial disease during this time. ETC testing from muscle biopsy material was traditionally sent to Clinical Laboratory Improvement Amendments (CLIA)-approved labs. Assays of ETC in each of the several approved centers used their own methodology and variability between laboratories was high in both enzymatic activity and internal standards^[18,19]^. Laboratories reported “normal” or “abnormal” based on their own methodology. Abnormality based strictly on published research diagnostic criteria of less than 20% of control values were not used by some laboratories. Despite some of the problems, ETC testing has inherited value of direct examining of oxidative phosphorylation (OXPHOS) capacity and, when performed under tightly controlled standards, has stood the test of time in diagnosis of mitochondrial disease in the genetic era^[20]^.

The genetic era of diagnosis began in the mid-1990s with the introduction of commercial availability in genetic testing for known mtDNA pathological variants causing human disease. Using this methodology, the choice of which gene to test was hypothesis- and phenotype-driven by clinician suspicion. Soon, adaptation of Sanger sequencing allowed genome mtDNA sequencing. Although an advancement in technology, Sanger sequencing is inadequate to detect some mtDNA mutations that occur in a small fraction of the total mtDNA molecules, heteroplasmic changes at lower than 15% (error of detection is ± 15%), or small deletions^[21,22]^. Even with this limitation, the gene discovery of novel pathological variants increased the numbers of confirmed mitochondrial disease patients. However, nuclear gene testing suffered from many genes causing similar phenotypes and many phenotypes induced by many distinct genes^[23]^. Guesswork on which gene to test made widespread testing unrealistic for most clinicians.

The advent of commercially available massively parallel sequencing or next-generation gene sequencing (NGS) exponentially increased the sensitivity of the diagnostic yield, but it illuminated the need for a better nosology of mitochondrial diseases. A few seminal works preceded the search for nuclear genes involved in mitochondrial diseases in the early 2000s. The first was the establishment of the protein spectrum of mitochondria function and structure, the MitoCarta. In two articles by the group from the Broad Institute using annotated genome sequences combined with tandem mass spectrometry and computation, the proteome was estimated to consist of ~1,160 proteins^[24,25]^. Today, further work has increased sensitivity; most think that the proteome contains about 1,500 proteins. Knowledge of these targeted gene products increased the yield of detected nuclear genes involved in mitochondrial disease. However, the complete compendium of the complete proteome remains unknown.

The next required factor in expanding the genetic detection of mitochondrial disease was commercial payment of genetic testing by insurance companies. Reimbursement has allowed widespread clinical testing and competitive pricing. NGS panels of genes became commercially available for suspected disease in 2010. Within the next three years, whole exome sequencing (WES) entered the commercial landscape. Gene panels began as limited spectrum of genes only involving nuclear- and mitochondrial-encoded ETC genes known to induce disease^[26]^. The effectiveness of this approach relied heavily on clinical acumen, analyte, muscle testing, and neuroimaging^[27]^. WES using NGS platforms soon moved into diagnostic testing. This has led genetic testing outside the predesignated MitoCarta platforms into an unbiased non-targeted “discovery” approach. Clinicians have now begun to use genetic testing to circumvent more expensive and tedious multiple biochemical analyte and muscle biopsy procedures for diagnosis. This has switched medical acumen to a “genetics first” approach^[28]^. Findings may be clinically relevant if the known variant occurs in a described disease gene, but, if the variant is of unknown significance, then functional validation of the change is needed. However, the discovery of previously described pathogenic variants in healthy individuals has muddied the waters on diagnosis^[29]^. Here, functional validation is vital for affirmation of pathogenicity, and a return to the past requiring skeletal muscle biopsy, analyte, and neuroimaging, with integration of functional protein alteration, animal models, and rescue cell and animal model systems, are needed for confirmation of variant pathogenicity^[30]^. Hence, one can understand why prevalence numbers for mitochondrial disease are low estimates.

Technology has now advanced to rapidly sequence the whole genome (WGS), which will expand diagnosis further. The current expense and lack of commercial insurance payment has greatly limited this technology from becoming mainstream. WGS can detect non-protein genetic factors that alter gene expression and hence modify disease or primarily cause disease. However, currently, WGS is mostly used in research labs and under certain circumstances, of urgent need. However, as technology improves, turnaround times shorten, databases enlarge, and costs reduce, WGS will likely significantly enhance our detection of disease-causing genetic alteration in the genome.

The rapid expansion of gene sequencing technology progressing from single gene, gene panels, whole exome, to now whole genome sequencing has produced substantial variability in the level of evidence for genotype to phenotype. The validation of each genetic variant within a gene supporting a gene-disease relationship is beyond the scope of this paper. Standard guidelines have been developed to support a gene-disease relationship and the subsequent framework to measure the strength of evidence of the gene-disease relationship. The NIH-funded Clinical Genome Resource was developed to serve this purpose^[30–32]^. The importance of this work to validate disease-causing gene changes is the high rate of variants of unclear significance highlighting the need for clinical and research input^[32]^. The culmination of this work is the ability for the clinician to provide the patient and family an accurate and timely diagnosis and to hopefully expand “precision” medicine treatments.

Nomenclature for mitochondrial diseases has been in flux due to changes in diagnosis and gene discovery. The most logical approach to nosology has not yet been formulated, but the methodology needs to include physiology, genetics, and clinical findings. The author leans toward the approach of physiological functions: proteins that directly produce ATP; mtDNA replication and maintenance factors; tRNA and rRNA biogenesis and transcription and translation factors; Fe-S cluster biogenesis enzymes, protein quality control, and import/processing proteins; membrane integrity components; mitochondrial dynamics proteins; and classic syndromes.

### Clinical manifestations of mitochondrial diseases

Mitochondrial diseases are clinically and genetically highly heterogeneous. Diseases can be inherited as autosomal recessive, dominant, or X-linked due to nuclear-DNA genetic variants and by maternal inheritance via mtDNA genetic variants. The presence of mitochondria in all nucleated cells, differing energy demand among body tissues, and expression of gene products produces a wide variety of symptoms [Table 2]. As discussed further below, the dual genome expression and interaction between genome products creates an additive spectrum of organ involvement in disease. In addition, there are multiple other influences on the age of onset, severity, and pattern of organ involvement and progression of disease. Mitochondrial disease expression is also confounded by environmental triggers, known as ecovariants^[33]^. Ecovariants are DNA sequences that remain silent unless exposed to environmental agents. For example, exposure of a patient with pathological variants in *polymerase gamma* (*POLG*) with the seizure medication valproic acid induces severe hepatopathy and certain nucleoside reverse transcriptase inhibitors induce lactic acidosis, pancreatitis, bone marrow suppression, myopathy, and peripheral neuropathy^[34,35]^. There are other DNA sequences that can act as modifiers by altering penetrance and expressivity of primary disease-causing pathological variants, but in and by themselves are silent^[36]^. For example, variants in the mtDNA sequence, m. 6480 G>A, m. 1281G>A, and m. 1539A>G, were found to increase penetrance and expressivity in patients with the Leber Hereditary Optic Neuropathy (LHON) pathological variant, m. 11778 G>A. There are also uncommon mtDNA-encoded variants that do not follow the “rules” of pathogenicity, but still cause disease^[37]^. The m. 8344 A>G change occurs in a region that is not strictly evolutionary conserved yet produces the syndrome of myoclonus, epilepsy with ragged red fibers (MERRF), one of the classic syndromes^[38]^. It is no wonder that there exists such a range of phenotypes. However, to confound the clinician, there are still disorders that seem to be restricted to one organ, such as disease due to certain mt-tRNA synthetases^[39]^. Furthermore, the lack of the full compendium of gene causing mitochondrial diseases can place the clinician in a conundrum of non-diagnostic disease.

Historical grouping of clinical features into certain syndromes has held true for the most part, but rarely does a phenotypic presentation have a direct correlation with any given biochemical, histopathology, enzymatic, or genetic findings^[40]^. Those mitochondrial syndromes that remain relevant today are likely the few diseases that, at the time of a clinical diagnosis, express organ involvement similarly in multiple individuals. As shown below, the confluence of symptoms, organ involvement, and genetic findings into syndromes is rare in mitochondrial disease. What is the linchpin to the range of disease phenotypes and genes is the unique physiology of mitochondrial function.

### Mitochondrial physiology

Mitochondria are dynamic organelles that undergo constant change in their structure as they move along large syncytial networks within the cell. The structure of this network is both state and tissue specific and, we think, intertwined with constant fission and fusion^[41]^. Mitochondria actively traverse the cytosol on dynein and kinesin tracks^[42]^. By unclear mechanisms, fission and fusion regulate mitophagy, mtDNA concentration, and subcellular organelle distribution by controlling recruitment of fission factor dynamin-related protein-1^[41,43]^.

**Figure 1.**
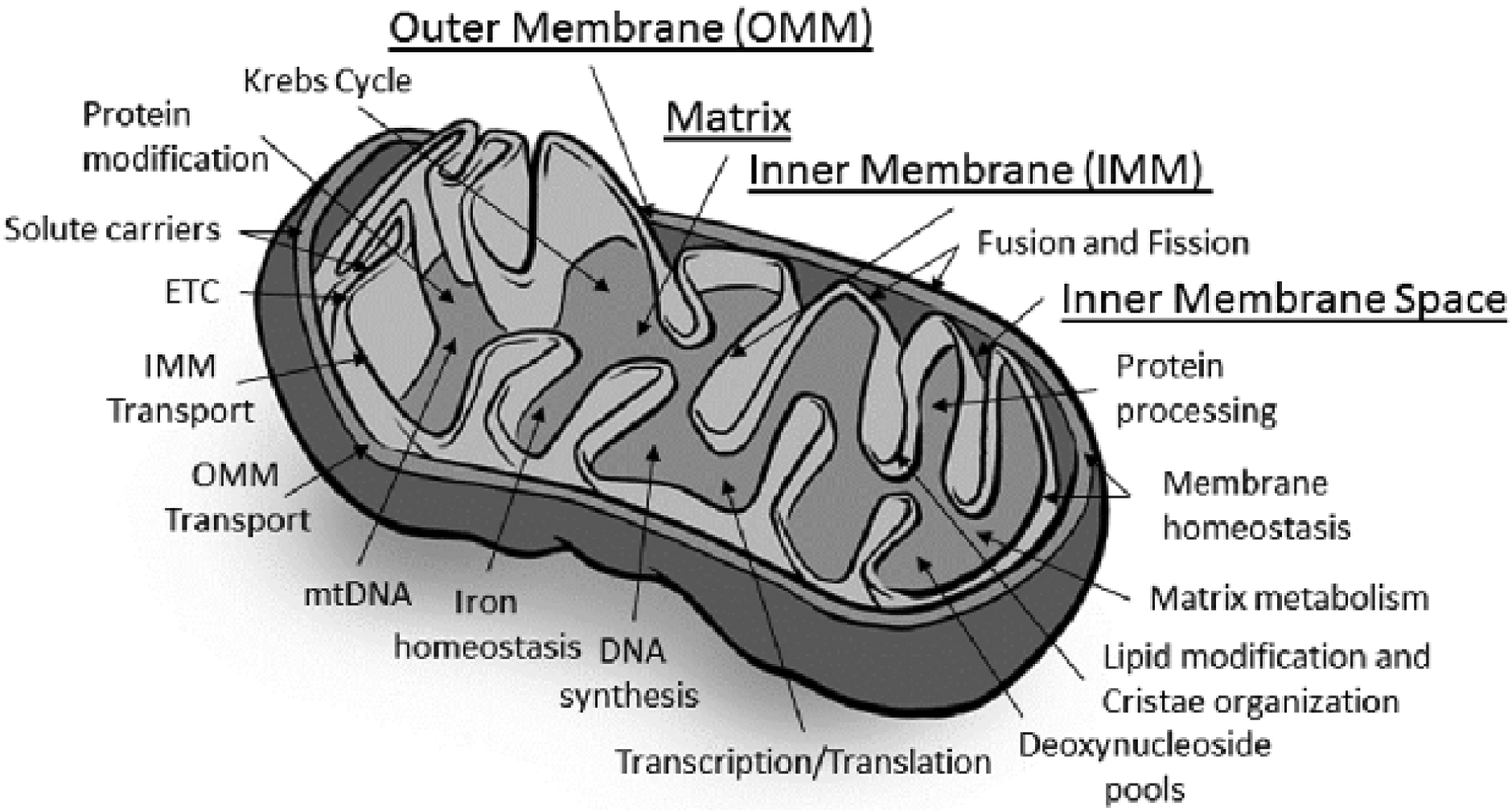
Structural elements and physiological processes. This figure represents the main structural elements and physiological processes carried out by the mitochondrion. Underlined labels represent the structural elements. Physiological functional processes are noted by arrows localizing where the processes occur within the mitochondrion. ETC: electron transport chain

The basis for the unique physiology of mitochondrion begins with its structure [Figure 1]. Mitochondria have two membranes, the inner mitochondrial membrane (IMM) and the outer mitochondrial membrane (OMM), separated by an intermembrane space that surrounds an inner matrix. The central hub of mitochondrial function, ETC, is embedded within the IMM, also called cristea. The ETC is composed of approximately 90 proteins, in five protein complexes, associated with reducing equivalents derived from the Krebs cycle and beta oxidation, NADH and FADH_2_. These reducing equivalents, in the form of electrons, are passed from Complexes I and II to coenzyme Q10. Complex III receives two electrons from coenzyme Q10 and passes them to Complex IV [also called cytochrome oxidase (COX)]. The reduction of oxygen into molecular water occurs at Complex IV (oxidative step). The shuttling of electrons from Complex I to Complex IV creates a proton gradient across the inner membrane into the inner membrane space. The resulting electrochemical charge across the IMM is the driving force of the proton flow back into the matrix through the pore of Complex V, generating ATP from ADP and phosphate (phosphorylation step). Once produced, ATP is exported from the matrix into the cell by the adenine nucleotide translocator (ANT) or used in the multiple molecular reactions within the matrix.

The process of OXPHOS is not completely efficient, and some unpaired electrons escape to form reactive oxygen species. The reactive oxygen species feed back to the nuclear compartment in the cell to help regulate nuclear control of mitochondrial function. Proton motive force is also intimately involved in calcium homeostasis in conjunction with the endoplasmic reticulum (ER), as well as apoptosis, solute and ion transport, protein import, and biosynthetic reactions within the matrix [Figure 1]^[44–46]^.

The matrix lies within the IMM. The inner membrane contains a special phospholipid, cardiolipin which is a four-tailed molecule. The structure of cardiolipin allows the inner membrane to take on a highly curved structure, inner membrane or cristae. Within the cristae lies the matrix, which contains hundreds of enzymes responsible for production of ATP and mitochondrial metabolism. The Krebs cycle and beta oxidation enzymes responsible for formation of NADH and FADH_2_ are found in the matrix. The matrix also contains enzymes needed for amino acid biosynthesis, and oxidation of several specific amino acids^[47]^. Each mitochondrion contains 2–10 copies of mtDNA, found within the matrix. In addition, enzymes involved in the urea cycle, DNA synthesis, metabolism of fatty acids, iron-sulfur biosynthesis, and functional proteins for apoptosis/autophagy and fusion are located within the matrix^[48,49]^.

### Mitochondrial genetics

The human mtDNA is a 16,569 kilobases (kb) closed-circular double-stranded molecule that encodes 13 polypeptides, 22 transfer RNAs (mt-tRNAs), and 2 ribosomal RNAs (mt-rRNAs)^[5]^. All nucleated cells contain between 100 and 10000 copies of mtDNA; higher numbers of mtDNA and mitochondria are regulated by energy demands. With extremely rare exceptions, all mtDNA is maternally-inherited. mtDNA is packaged in protein-DNA complexes, nucleoids, which also contain the machinery required for mtDNA replication, transcription, repair, packaging, and stability^[50,51]^. The unique nature of mitochondrial inheritance and multiple mitochondrion per cell with each mitochondrion having many mtDNA molecules, has produced several unique physiological features. Homoplasmy is a term describing when all mtDNA molecules contain a relevant gene in question has the identical sequence. Heteroplasmy is the term where two or more variant populations of a particular mtDNA sequence exist within one cell. The heteroplasmic level/load of a variant can vary between tissues in a single individual, complicating disease detection^[52]^. Not all mtDNA variation leads to disease: some variants are neutral, others may confer a selective advantage to the cell or organism, and still others are risk factors for disease^[53]^. When disease causing, pathogenicity is determined by the nature of the mutation and relative abundance or heteroplasmy level^[54]^. The relative amount of variant heteroplasmy that produces disease is named threshold, and it can vary from tissue to tissue. This variation of levels between tissues types can produce a mosaic of organ dysfunction within an individual^[52]^. Most pathological variants are considered “recessive” because high levels of heteroplasmy (threshold) are required to manifest cellular defect or clinical phenotype. However, heteroplasmy cannot always explain the phenotype variability seen. For example, in LHON patients, being male is a predictor for disease expression, even though both females and males in the same family express 100% homoplasmy of the mtDNA pathological variant^[7]^.

### Electron transport chain disorders

The 13 mtDNA encoded proteins are essential proteins encoding subunits of the ETC, Complexes I, III, IV, and V. The subunits encoded by mtDNA are all essential hydrophobic components of ETC (OXPHOS) housed within the inner membrane. There are another 79 subunits encoded by nuclear DNA, including all subunits of Complex II^[55]^. Pathological variants have been reported in all 13 mtDNA structural genes, as well as mt-tRNA and mt-rRNA genes^[56,57]^.

Complex I (NADH: ubiquinone oxidoreductase) is the largest component of the ETC system, comprising 45 subunits. Electrons, in the form of NADH, enter ETC at Complex I. Complex I is involved as part of the supercomplex consisting of one Complex I, two Complex III, and one Complex IV unit aggregates, which power the production of ATP at Complex V. There are seven mtDNA-encoded subunits of Complex I combined with seven nuclear-encoded subunits that are responsible for the catalytic activity of Complex I. Each of these subunits has validated pathological variants causing disease [Tables 3 and 4]. The other 31 supernumerary subunits have roles that have not been completely elucidated. However, pathological variants have been determined in 13 of these latter subunits, suggesting significant roles in Complex I function. There are at least 15 assembly factors, of which 11 have been associated with disease^[58–62]^. Isolated Complex I deficiency represents the most common cause of single ETC-induced disease, accounting for 30% of them in the pediatric population^[56]^. Leigh syndrome is the most frequent expression of Complex I defects^[63,64]^. Studies have demonstrated that the majority of Complex I pathological variants are private and nonrecurring^[65]^. The clinical phenotype of Complex I dysfunction are heterogeneous, often including leukodystrophy and/or cardiomyopathy and associated with early death.

Complex II or succinate dehydrogenase serves two functions in mitochondrial metabolism. Reducing substances created in the Krebs cycle, in the form of FADH_2_, are passed from succinate dehydrogenase to ubiquinone as succinate is formed into fumarate. Complex II is located in the matrix associated with the IMM. Complex II is composed of two catalytic subunits, SDHA and a ferrous sulfate containing subunit, SDHB. The catalytic subunits are anchored to the IMM by the subunits, SDHC and SCHD. There are two known assembly proteins, SDHAF1 and SDHAF2. All four protein subunits and the two assembly proteins are nuclear DNA-encoded.

Disease-causing recessive variants in Complex II structure or assembly are an uncommon etiology of mitochondrial disease, accounting for only 2%−8% of mitochondrial cases^[66]^. The most common subunit involved in Complex II-derived disease is in SDHA, with less than 35 patients reported^[67]^. The two main phenotypes associated with Complex II-encoded genes, *SDHA, SDHB, SDHD*, and the assembly factor *SDHAF1* are associated with progressive encephalopathy leukodystrophy, Leigh syndrome, and/or cardiomyopathy^[68,69]^. Heterozygous mutations in *SDHB* and *SDHD* have also been linked to pheochromocytoma-paraganglioma syndromes^[70]^. Mechanism of isolated tissue localization of the latter syndromes remains unknown.

Complex III (ubinquinol-cytochrome c oxidoreductase) transfers electrons from ubiquinol to cytochrome b and then to cytochrome c. There are 11 structural subunits, 2 heme groups, and the Rieske iron-sulfur protein. Pathological recessive variants in one mtDNA-encoded gene, *MT-CYB*; four nuclear encoded subunits, *CYC1*, *UQCRB*, *UQCRC2*, and *UQCRQ*; and five assembly factors, *BCS1L*, *LYRM7*, *TTC19*, *UQCC2*, and *UQCC3*, have been reported to give rise to disease^[71]^. Recessive variants have been associated with developmental delay, encephalopathy, hepatopathy, renal tubulopathy, exercise intolerance, and muscle weakness.

Pathological variants in the *BCS1L* gene are the most common cause of Complex III deficiency. The BCS1L protein facilitates the last step of Complex III assembly, the insertion of the Rieske iron-sulfur subunit^[72]^. Pathological variants produce two phenotypic syndromes: syndrome of growth retardation, aminoaciduria, cholestasis, iron overload, and early death (GRACILE) and Bjorstand syndrome (abnormal flattening and twisting of hair shafts and hearing problems). As with other mitochondrial diseases, there are a range of phenotypes with variants in the *BCS1L* gene ranging from adults with aminoacidura, seizures, sensorineural deafness, and learning difficulties to infants with early death^[73]^.

COX or Complex IV is the terminal step of the respiratory chain involved in accepting electrons from cytochrome c to reduce oxygen to water. COX is composed of 14 subunits; 3 are mtDNA-encoded genes and 11 are nuclear-encoded genes. Variants in eleven subunits are associated with isolated Complex IV deficiency: three are mtDNA-encoded, *MT-CO1*, *MT-CO2*, and *MT-CO3*, and eight are nuclear-encoded, *COX411*, *COX 412*, *COX5A*, *COX6A1*, *COX6B1*, *COX7B*, *COX8A*, and *NDUFA4*^[74]^. There are at least 26 known assembly/ancillary proteins with unknown numbers of other proteins, with ten associated with Complex IV deficiency: *CEP89*, *COX14*, *COX20*, *COA3*, *COA7*, *COA8*, *PET100*, *PET117, TACO1*, and *SURF1*^[75]^. Remarkably, *SURF1* pathological variants account for the most common genetic etiology of Leigh syndrome, even though over 79 genes have been documented to cause this disorder^[76]^. One patient with variants in *COX8A* has been reported to induce Leigh syndrome, but why the other 24 assembly/ancillary proteins have not been shown to produce this disease is unclear^[77]^. There has been a milder-form of Charcot-Marie-Tooth syndrome caused by *COX6A1* pathological variants^[78]^. The protein APOPT1/COA8 has been recently shown to protect COX assembly from oxidation-induced degradation^[79]^. Biallelic *APOPT1/COA8* variants have been shown to cause COX deficiency and cavitating leukoencephalopathy^[80]^.

ATP synthase or Complex V is the final step in ETC, the phosphorylation of ADP to ATP. There are two functional domains, F1 and Fo. The F1 domain comprises five different subunits and is situated in the matrix. Fo domain contains six subunits, which are associated with five accessory subunits^[81]^. The only two mtDNA-encoded subunits are found in the Fo domain, *ATP6* and *ATP8* genes^[5]^. Pathological variants in the *ATPase 6* gene have been described as inducing Leigh syndrome^[82]^. ATPase 6 dysfunction encompasses phenotypes of neuropathy, ataxia, and retinitis pigmentosa and Leigh syndrome based on heteroplasmy^[83]^. Additional related phenotypes include leukencephalopathy, seizures, and renal disease^[84]^. The structural gene, *ATP5A1*, has been described to induce phenotypes ranging from severe infantile encephalopathy and early death, to a patient with polyneuropathy and mild mental retardation^[81,85]^. A common nuclear-encoded variant disease is encoded by *TMEM70*, an assembly factor of Complex V^[86]^. The *TMEM70* gene produces a phenotype of neonatal onset, cardiomyopathy, facial dysmorphism, lactic acidosis, and 3-methylglutaconic aciduria. Variants in *USMG5* induce loss of Complex V dimerization and loss of cristae curvature at the apex of cristae, demonstrating dimerization and structural architecture are required for full activity of Complex V^[87]^. *USMG5* encodes a small protein that is a supernumerary subunit of Complex V that is required for Complex V dimerization and ATP synthetase activity^[88]^.

One of the common mitochondrial syndromes is Leigh syndrome [Table 1]. Approximately 10% of Leigh syndrome patients have pathological variants in Complex V, at position m. 8993A>G^[89,90]^. The other genetic etiologies of Leigh syndrome represent both mtDNA-encoded and nuclear DNA-encoded genes. Pathological variants in mtDNA-encoded genes include almost all Complex I subunits, ND1, ND2, ND3, ND4, ND4L, ND5, and ND6; Complex IV subunit, COX III; and the mt-tRNA-encoding genes for lysine, valine, leucine, and tryptophan^[64]^. The most common nuclear-encoded gene inducing Leigh syndrome is *SURF1*, which is an assembly factor for Complex IV^[64,91]^.

The most common mitochondrial disease in infants and children is Leigh syndrome. Onset is typically between 3 and 12 months, but can range from birth to adulthood^[89,92]^. Disease presentation usually begins in the context of a viral illness or infection, after normal early development. Presentation of feeding issues, nystagmus, and/or optic atrophy heralds the disorder. Progression of disease is noted by ataxia, eyelid ptosis with ophthalmoparesis, dystonia, respiratory problems, and gastrointestinal abnormalities become expressed. The median time from disease onset to death is approximately 1.8 years, with over 50% of patients dying due to respiratory complications^[89]^.

The hallmark findings of bilateral symmetric lesions within the brainstem and basal ganglia structures define Leigh syndrome^[13]^. Pathologically, these lesions consist of spongiform and necrotic tissue. The advent of MRI imaging has replicated the changes and has essentially replaced the need for pathological confirmation^[93]^. There have been strict clinical criteria for identification of Leigh syndrome involving neuroradiological/pathological findings, intellectual and motor developmental delay, and elevated serum or cerebrospinal fluid lactate^[90]^. Although the genetic and phenotypic landscape of mitochondrial diseases has exponentially exploded, Leigh syndrome represents one of the anchoring definitions of mitochondrial disease expression.

Coenzyme Q10 (CoQ10) or ubiquinone is a 1,4-benzoquinone molecule located within the inner membrane of the human mitochondrion. It functions within the ETC as an electron shuttle from Complexes I and II to Complex III. The compound also has properties to act as an antioxidant, it controls mitochondrial uncoupling, it is required for pyrimidine nucleoside biosynthesis, and it regulates apoptosis^[94,95]^. There are 15 proteins/enzymes required for CoQ10 synthesis, of which nine are associated with disease. Each of the CoQ10 synthesis genes (*COQ2*, *COQ4*, *COQ6*, *COQ7*, *COQ8A*, *COQ8B*, *COQ9*, *PDSS1*, and *PDSS2*) has been shown to give rise to primary CoQ10 deficiency^[96]^. There are also secondary deficiencies of CoQ10 due to variants in *APTX*, *BRAF*, and *ETFDH*, which create a CoQ10 deficient state not directly involved in CoQ10 synthesis^[95]^. However, extensive genetic testing has failed to uncover genetic etiologies of some patients who have low CoQ10 levels and compatible disease^[95]^. The phenotype of CoQ10 deficiency ranges widely, but mostly involving the renal, cardiac, eye, hearing, muscle, and central nervous system. A common abnormality is a steroid-resistant nephrotic syndrome associated with *COQ2*, *SOQ6*, *COQ8B*, and *PDSS2* variants. All variants have central nervous system involvement with encephalopathy and many patients also express seizures and ataxia with comorbid myopathy. Age of onset is quite variable, from birth to the seventh decade of life. Searching for CoQ10 deficiency is critical in patient management, as high-dose oral supplementation can be helpful for those with primary and secondary deficiency.

Cytochrome c is a water-soluble 13 kilodalton (kDa) heme protein that shuttles electrons between Complex III to Complex IV. It is bound to cardiolipin in the IMM. There are two genes responsible the structure of the protein. *HCCS* is an X-linked gene encoding the holocytochrome c-type synthetase that covalently binds the prosthetic heme group to apocytochrome c^[97]^. There is some evidence that *HCCS* variants may give rise to microphthalmia with linear skin defects syndrome associated with segmental monosomy of the Xp22 region^[98]^. The other nuclear-encoded gene involved in synthesis of cytochrome c is *CYCS*, which encodes the apocytochrome c. A loss of function deletion in the *CYCS* gene has recently been linked to non-syndromic thrombocytopenia in a Japanese family^[99]^. To date, it remains unclear how cytochrome c is transported into the mitochondria as no mitochondrial leader sequence has been found. Cytochrome c is also intimately involved in the primary apoptotic pathway. When the cell detects DNA damage, metabolic stress, or the presence of unfolded proteins, the intrinsic apoptotic pathway is triggered, and cytochrome c is released into the cytoplasm and triggers programmed cell death^[100]^.

### Disorders of mtDNA, replication, and maintenance

#### Rearrangements in mitochondrial DNA

Sporadic, mostly non-inherited group of diseases derived from a single large nucleotide deletion in sizes from 1.3 to 7.6 kb produce three classic mitochondrial syndromes^[101]^. The most common deletion is approximately 5 kb in length. The location of this deletion spans the *ATPase 8* gene to the *ND5* gene, and both genes are flanked by a perfect 13-base pair-directed nucleotide repeat^[102]^. In a large study of 67 patients, 40% had a 5-kb deletion in muscle samples. In those patients less than six years of age, 85% had various sizes of deletion other than the 5 kb seen in older patients. The location varied within the mtDNA and was higher in heteroplasmy in this younger population. Further studies have shown that, even though most deletions occur sporadically during early development, the identical deletion is found in all affected tissues^[103]^. Affected patients are always heteroplasmic and a fraction of > 60% mutant load is required to impair mitochondrial protein translation^[104]^. The exact mechanisms of producing single large deletions during development remain unclear. Recently, an inherited autosomal recessive variant in the mitochondrial single-stranded DNA-binding protein 1 (*SSBP1*) has been shown to produce a single large mtDNA deletion^[105]^. Duplications of mtDNA have not, to date, been reported to cause disease with the exception of a single case report^[106]^.

Single large deletions in mtDNA can induce one of three classic clinical syndromes: Pearson syndrome, Kearns-Sayre syndrome, and CPEO [Table 1]. Some patients with large deletions also demonstrate Leigh syndrome, hearing loss, myopathy, retinitis pigmentosa, diabetes, pancreatitis, cardiomyopathy, and ataxia.

Pearson syndrome onset is during infancy presenting as refractory sideroblastic anemia with vacuolization of bone marrow precursor cells and co-morbid pancreatic failure^[107]^. On genetic testing, these children have a single mtDNA deletion; as with the other syndromic single deletion disorders, the most common size of deletion is 5 kb. The vast majority of patient have a de novo deletion with maternal inheritance very rare. Interestingly, the deletion is found in most tissues, and the most logical assumption is that this event is very early in embryogenesis^[108]^. Since most tissues are affected, blood leukocyte testing is routinely positive. The multiple tissue involvement creates a multisystem disease with short stature, proximal renal tubulopathy, skin rash, liver failure, and chronic diarrhea. The clinical course is progressive with most having death during childhood. Those patients who survive childhood develop Kearns-Sayre syndrome. The progression of the disease has a high correlation with size of deletion, heteroplasmy level in muscle, and location of mutation in the mtDNA^[109]^.

Kearns-Sayre syndrome is classically a triad of onset before 20 years of age, CPEO, pigmentary retinopathy, and at least one of the following: cardiac conduction block, cerebrospinal fluid protein concentration greater than 0.1 g/L, and/or cerebellar ataxia^[110]^. Other frequent clinical findings include short stature, cognitive impairment, sensorineural hearing loss, renal tubular acidosis, seizures, progressive myopathy, and endocrinopathies. The retina displays a “salt and pepper” retinopathy of the posterior fundus and does not produce visual field defects. MRI of the brain usually demonstrates cerebral and cerebellar atrophy and leukodystrophy^[93]^. Even though only approximately 57% of patients with Kearns-Sayre syndrome have cardiac conduction defects, 20% of these patients die of sudden cardiac death^[111]^. Due to selective elimination of deleted mtDNA in proliferating leukocytes, the older age of Kearns-Sayre presentation requires sampling from muscle tissue, and it is required for accurate heteroplasmy^[112]^. Accuracy in heteroplasmy determination combined with gene deletion containing at least one of the *MT-CO1*, *MT-CO2*, or *MT-CO3* genes is related to disease progression^[108]^.

CPEO generally develops in mid-adulthood, but a significant correlation exists between size of deletion, heteroplasmy, and age of onset^[113,114]^. Early age of onset is associated with small deletion size and higher heteroplasmy. Characteristically, there is eyelid ptosis and a slowly progressive paralysis of the eye muscles leading to impaired eye movements. As paralysis continues, often compromised upgaze is the first symptom noted. Muscle weakness, sensorineural hearing loss, diabetes mellitus, proximal muscle weakness, and progressive dysphagia are often co-morbid symptoms. In both CPEO and Kearns-Sayre syndromes, hearing loss and diabetes mellitus can precede onset of muscle involvement^[115]^.

Multiple mtDNA deletions can also give rise to CPEO. The etiology of multiple mtDNA deletion are caused by pathological variants in nuclear DNA-encoded genes (see section below). In a large cohort of 136 patients with a single large mtDNA deletion, none of the patients had a polyneuropathy^[115]^. However, other than the presence or absence of peripheral neuropathy, the clinical phenotype of CPEO is similar between single and multiple deletions in mtDNA^[113]^. There has recently been a patient who has a single large 5-kb mtDNA deletion caused by a nuclear-encoded gene involved in mitochondrial DNA replication, *SSBP1*^[105]^. This gene’s product binds and protects single-stranded DNA during mtDNA replication. Phenotypically, this child presented with bone marrow failure and infantile anemia, similar to Pearson syndrome; and short stature, ptosis, ophthalmoplegia, retinal dystrophy, sensorineural hearing loss, and multiple endocrine deficiencies, similar to Kearns-Sayre syndrome. He also developed metabolic strokes. A dominant variant in *SSBP1* has been reported to induce optic atrophy and foveopathy^[116]^.

#### Mitochondrial DNA replication and maintenance

The replication of mtDNA is continuous throughout the cell cycle and even in those cells not undergoing active replication. The apparatus needed for replication is exclusively encoded by nuclear genes. The requirement of multiple copies of mtDNA per mitochondrion and numerous mitochondria per cell underscore the importance of mechanisms of ongoing mtDNA integrity and stability. The constant synthesis of mtDNA requires a balanced pool of precursor nucleotides ready for incorporation by the replisome machinery into new DNA molecules^[117]^. The precursor nucleotide pools are provided by salvage pathways and import from the cytosol by specific transporters. Single copies of mtDNA are packaged into nucleoprotein complexes, named nucleoids within the IMM. The precise location of mtDNA replication is thought to occur within the nucleoid complex, as, in experiments with purification of necleoids, POLG, Twinkle, and mtSSB are found^[118]^. Each nucleoid complex has multiple copies of mitochondrial transcription factor A (TFAM), which is involved in mtDNA compaction and responsible for replication and transcription within mitochondria^[119]^. When TFAM is at high concentrations, replication and transcription is blocked, but, when compaction is mild, both processes can continue. Enriched nucleoid preparations also contain components of transcription, RNA helicases, RNA-binding proteins, quality control proteases, RNA processing proteins, and a subset of mitochondrial ribosomal proteins^[120]^. Newly replicated nucleoids are likely coupled to mitochondrial fission at ER contact sites^[121]^. Mitochondrion constantly undergo fusion and fission, termed mitochondrial dynamics^[41]^. The central role of nucleoid function, in addition to mtDNA replication and mitochondrial transcription and translation, is still being defined. However, the importance of the integrity of the nucleoid can be demonstrated when deficiencies of mtDNA replication and editing, repair, fusion, fission, and alterations in the balance within nucleotide pools result in mtDNA depletion, multiple mtDNA deletions (and single large deletion), and age specific mtDNA point mutations producing disease [Tables 3 and 4].

#### Mitochondrial DNA replication apparatus

The center piece of mtDNA replication revolves around a specialized mtDNA polymerase, POL gamma. The polymerase is a heterotrimer composed of one 140-kDa catalytic subunit encoded by *POLG* (also reported in the literature as *POLG1* and *POLGA*) and a homodimeric processing subunit composed of two p55 accessory proteins encoded by *POLG2*. POL gamma is responsible for the replication of mtDNA, proofreading, and repair of replication mistakes^[122]^. The replisome consists of POL gamma, one POLG subunit and two POLG2 subunits, helicase Twinkle (encoded by *TWNK* formerly named *C10orf2*), mitochondrial topoisomerase I, mitochondrial RNA polymerase, RNase H1 (encoded by *RNASEH1*), and mitochondrial genome maintenance exonuclease 1 (MGME1 encoded *MGME1*). Other proteins involved in mtDNA replication are mitochondrial single-stranded DNA binding protein 1 (mtSSBP1), DNA ligase III, DNA helicase/nuclease 2 (DNA2 encoded by *DNA2*), and RNA and DNA flap endonuclease (*FEN1*)^[123]^. POLG-related disease depends on the location of the pathological variant within the gene and likely unknown environmental and epigenetic factors. The intermixing of gene variants and other factors largely determine the clinical presentation and course of disease from the severe infantile onset of hepatocerebral disorder, Alpers-Huttenlocher syndrome, to adult onset CPEO^[33]^. The most severe spectrum of POLG disease is associated with mtDNA depletion and childhood onset. The milder disorders have onset in early-to-late adulthood and associated with multiple mtDNA deletions. Variant changes within the *POLG* gene can determine mode of inheritance. Variants found in certain sites within the polymerase region produce autosomal dominant or recessive disease, but variants in the linker and exonuclease regions produce autosomal recessive disorders. Furthermore, the phenotype and genotype remain blurred as both autosomal dominant and recessive variants induce CPEO. However, in a large cohort of CPEO patients, all had either a single large mtDNA deletion or multiple small mtDNA deletions^[113]^. However, not all patients with variants in genes responsible for multiple mtDNA deletions have CPEO^[117]^. The reasons for this remain unknown. However, unlike single large mtDNA deletion-induced CPEO, those nuclear-encoded genes giving rise to CPEO and multiple mtDNA deletion express sensory neuropathy^[124]^. Medically refractory seizures early in life is associated with mtDNA depletion, autosomal recessive inheritance, and mostly associated with Alpers-Huttenlocher syndrome^[125]^. The mechanism of valporic acid-induced hepatopathy in Alpers-Huttenlocher syndrome is not understood^[34]^.

Some genes give rise to both mtDNA multiple deletions and mtDNA depletion, while others do not. The exact mechanism of why this happens is not clear. Mutations in the 5’−3’ DNA helicase *TWNK* induces both autosomal recessive and dominant disease. The protein product of *TWNK*, twinkle, acts as the DNA and RNA helicase. The dominant form of the disease causes an adult onset CPEO with myopathy and multiple mtDNA deletions^[126]^. The autosomal recessive disease is expressed as an mtDNA depletion syndrome presenting with severe epileptic encephalopathy^[127]^. Other variants in *TWNK* induce a recessive syndrome found in the Finnish ethnic group that produces infantile onset spinocerebellar ataxia, with mtDNA depletion found in the liver and brain^[128]^. SSBP1 is required to stabilize single-stranded mtDNA and stimulates DNA synthesis by POLG. As described above, dominant pathological variants in *SSBP1* have recently been shown to induce optic atrophy, hearing loss, and foveopathy with mtDNA depletion, and recessive variants express features of Pearson, Kearns-Sayre, and Leigh syndrome^[105,116,129]^.

Several of the replisome gene products are involved in the maturation of newly synthesized mtDNA strands and have been implicated in mtDNA repair. *MGME1* produces a single-stranded DNA flappase involved in processing of 5’ mtDNA ends generated during replication. Compromise in this processing protein produces multiple mtDNA deletions with disease onset from childhood to adulthood. Disease is expressed by CPEO, muscle weakness and wasting, and respiratory muscle weakness^[130]^. *DNA2* and *RNASEH1* products are found both in the nucleus and mitochondrion, and both products are involved in mtDNA repair in long-patch base-excision repair. Variants in these genes produce CPEO and proximal muscle weakness^[130,131]^. Onset of disease is distinct, variants in *DNA2* present in childhood while *RNASEH1* presents during adulthood^[131]^.

#### Defects in the control of mitochondrial deoxyribonucleoside pools

The requirement for constant mtDNA replication requires an adequate and balanced pool of deoxyribonucleoside triphosphates (dNTPs) and deoxyribonucleoside 5’triphosphate precursors. Multiple mtDNA deletions and/or depletion have been associated with pathological variants in nine nuclear encoded genes involved in the balance of dNTP pools^[12,117,132]^. Synthesis of dNTPs occurs by the de novo pathway that is cell cycle dependent or by the cell cycle independent salvage pathway. The salvage pathway is uniquely important for dNTPs in post-mitotic cells such as neurons and muscle cells. Alterations in the balance of nucleotide pools lead to increased mutagenesis or mtDNA deletions. There are six genes that control intramitochondrial dNTP giving rise to disease: *SUCLA1*, *SUCLG1*, *TK2*, *SAMHD1, ABAT*, and *DGUOK*. The three other genes that control dNTP pools also induce disease: *TYMP*, *RRM2B*, and *GUK1*.

mtDNA diseases arising from dysregulation of dNTP pools mostly induce mtDNA multiple deletions, however some also induce mtDNA depletion. Depletion-induced diseases are more severe and earlier in onset compared to multiple deletion syndromes. For example, TK2-induced mtDNA depletion usually presents before the age of two years with myopathy, feeding difficulty, hypotonia, and within a few years respiratory failure^[133]^. Multiple deletions induced by TK-2 presents later in life with CPEO and proximal muscle weakness^[134]^. *DGUOK* is involved in the purine nucleoside salvage pathway^[135]^. Patients with variants in *DGUOK* producing mtDNA depletion present with early onset liver dysfunction and subsequent hepatic failure with co-morbid neurodevelopment delay, abnormal eye movements, and hypotonia^[136]^. Variants in the *DGUOK* inducing mtDNA deletions produce disease with adult onset CPEO, myopathy, and Parkinsonism^[137]^. Both *SUCLA1* and *SUCLG1* have similar phenotypes with infantile onset and mtDNA depletion^[138]^. The protein products are involved in the citric acid cycle and stabilize enzymes involved in mtDNA nucleotide pools. Phenotypically, *SUCLA1*/*SUCLG1* variants induce early childhood hypotonia and subsequently develop muscle atrophy with psychomotor delay. Early patient death occurs in the *SUCLG1* variants, but those with the *SUCLA1* variants survive into their twenties. ABAT is the main enzyme responsible for catabolism of the neurotransmitter gamma-aminobutyric acid and regulates mitochondrial nucleoside salvage. Variants in *ABAT* induces infantile spasms, significant developmental delay and hypotonia, and mtDNA depletion^[139]^. SAMDH1 is a triphosphohydrolase converting dNTPs to deoxynucleosides and interfaces with DGUOK to cause mtDNA depletion^[140]^. Thus far, pathological variants in *SAMDH1* have not been described.

Variants in ribonucleotide reductase, p53-R2 subunit (RRM2B) can cause mtDNA depletion and severe neonatal/infantile myopathy with some patients developing tubulopathy, seizures, and respiratory compromise with death before one year of age^[141,142]^. A milder adult onset type with mtDNA deletions presents with CPEO^[143]^. RRM2B is the main regulator of nucleotide pools in the cytoplasm and is likely key in maintaining dNTP pools for mtDNA synthesis^[144]^. The cytoplasmic enzyme thymidine phosphorylase encoded by *TYMP* regulates pyrimidine deoxyribonuclesidases, thymidine, and deoxyuridine. Loss of TYMP function induces systemic accumulation of thymidine and deoxyuracil in serum and tissues with changes in mtDNA stability and, consequently, the mitochondrial syndrome of mitochondrial neurogastrointestinal encephalopathy (MNGIE)^[145,146]^. Both mtDNA multiple deletions and mtDNA depletion have been described and related to region of organ dysfunction within the GI tract, the small intestine with depletion, and upper GI tract multiple deletions^[147]^. The onset of MNGIE is late adolescence, but it can occur at any age (range 5 months to 35 years)^[147]^. The cardinal features of MNGIE are ptosis, ophthalmoparesis, leukoencephalopathy, peripheral neuropathy, and severe gastrointestinal dysmotility with cachexia [Table 1].

#### Mitochondrial transcription

The mitochondrial genome has 22 genes for tRNA needed for transcription within the IMM anchored nucleoid. Transcription of mRNA occurs from both heavy and light strands of mtDNA as large polycistronic precursor mRNA molecules from each strand. Historically, each strand of the mtDNA duplex is labeled by the number of guanine nucleotides (H-strand for heavy) and cytosine residues (L-strand for light) that correlated with buoyant density. Both strands express mtDNA genes differentially, the H-strand consists of 12 of the ETC subunits and 2 of the tRNA genes, while the L-strand produces the other mtDNA genes^[5]^. There are three essential components of transcription initiation, mitochondrial transcription factor A (TFAM) and B (TFB2M), and the single mitochondrial RNA polymerase (POLRMT). Initiation of transcription begins by TFAM binding just 10–15 base pairs upstream from the start site, PSP on the L-strand, and HSP 1 promoter site with unwinding of the DNA^[148]^. Then, TFAM attracts POLRMT and TFB2M, and subsequently both of the latter bind to TFAM^[149–151]^. Both strands of mtDNA are transcribed simultaneously with termination performed by the mitochondrial termination factor 1 (MTERF1)^[152]^. The mechanism of how MTERF1 terminates transcription of the polycistronic strand remains unclear, but data indicate that it actively terminates L-strand transcription, with indirect stoppage of the H-strand^[153]^.

The importance of proper transcription and translation of the 13 mtDNA-derived subunits for proper functioning is the finding that all 22 mt-tRNAs encoded by mtDNA have pathological variants inducing disease. There are 45 reported confirmed pathological point variants in MITOMAP (http://www.mitomap.org/MITOMAP) with over 200 variants reported as possible or likely pathogenic variants. The pathological variants in mt-tRNA producing disease involve either negative effect on biogenesis and functioning of tRNAs after their transcription; including processing, post-transcriptional modification, aminoacylation, association with mt EF-Tu, and/or interactions with mitoribosome during translation^[154,155]^. To completely eliminate tRNA function directly, the pathological variant would have to alter one of the anticodon bases or the discriminatory bases that are critical for codon recognition and aminoacylation, which is a rare occurrence and likely would lead to incompatibility with life. The majority of variants altered tertiary structure of the tRNA, efficiency of 3’ processing, and elongation factor binding^[155]^.

One of the most common mtDNA-encoded disorders is the syndrome, mitochondrial encephalopmyopathy, lactic acidosis, and stroke-like episodes (MELAS). Over 80% of all patients have a pathological variant m. 3243A>G in the tRNA^Leu^ gene, *MT-TL1*^[156,157]^. There are at least five other pathological variants in the *MT-TL1* gene as well as other mt-tRNA genes, protein-encoding genes, and large deletions have been described as inducing MELAS^[157]^. The invariant clinical criteria for diagnosis are: stroke-like episodes before the age of 40 years, encephalopathy characterized by seizures and/or dementia, and mitochondrial myopathy, as evidenced by lactic acidosis and/or ragged red fibers. The diagnosis is confirmed if there are at least two of the following: (1) normal early psychomotor development; (2) recurrent headaches, and (3) recurrent vomiting episodes^[158]^.

#### Mitochondrial translation

The essential backbone of tRNA and rRNA for protein synthesis or translation, in addition to the 13 mtDNA-encoded subunits in the ETC, are found in the mitochondrial genome, 2 rRNA subunits and 22 tRNAs. There are at least 80 nuclear-encoded proteins required for proper mt-ribosome function^[159,160]^. There are at least another 80 nuclear DNA-encoded products needed for translation, mRNA processing and maturation, and protein synthesis of the 13 ETC subunits^[161]^. After synthesis in the matrix, proteins are inserted into the IMM and assembled into the ETC protein complex.

The translation process can be divided into initiation, elongation, termination, and recycling. A full description is beyond the scope of this paper, but recently reviewed by Ott *et al*.^[162]^. Briefly, after the dissociation of the mitoribosome complex, the process of translation is started by the binding of the charged fMet-tRNA to the small rRNA subunit. This binding causes the large ribosomal subunit to bind, the completed monosome is formed, and elongation begins. The elongation process continues until the stop codon is reached and the completed polypeptide is released. The two ribosomal subunits dissociate and the mRNA and deacylate mt-tRNA are recycled.

Nine of the 80 nuclear-encoded genes in the mitochondrial ribosomal proteins and one of the two rRNA-encoded genes have been reported to induce disease: *mtRNR1*, *ERAL1*, *MRPS7*, *MRPS16*, *MRPS22*, *MRPS23*, *MRPS34*, *MRPL3, MRPL12*, and *MRPL44*^[162–164]^. These disorders range from isolated primary ovarian failure to multisystemic involvement of global development, hepatopathy, cardiomyopathy, kidney disease, neurosensory hearing loss, and Leigh syndrome. Death can be during infancy or survival can last multiple decades. When a patient carrying the m. 1555 A>G and m. 1494 C>T variants in the mtDNA-encoded small ribosome, *mtRNR1*, is exposed to aminoglycosides, hearing loss occurs; this effect is profound that even only a single dose results in hearing loss^[165,166]^. These ecovariants lie silent unless the patient is exposed to an aminoglycoside antibiotic.

#### Mitochondrial mt-tRNA and mt-mRNA processing and stabilization

The complete overview of mt-tRNA processing and stabilization is beyond the scope of this article but can be found in the review by Hallberg and Larsson^[167]^. As the transcript is synthesized, the mt-tRNAs are processed first at the 5’ end, and then in a subsequent step at the 3’ end. The 5’ end of the mt-tRNA is released by the RNase P-complex (RNase P). The RNase P complex is composed of three proteins, MRRP1, MRRP2, and MRRP3. Two of the RNase P components have a methyltransferase activity and this subcomplex is first to bind the 5’ end of the mt-tRNA, likely then followed by the MRRP3 protein, and cleaves the primary transcript at the 5’ end. Alterations in both *MRRP1* and *MRRP2* have been shown to cause disease. *MRRP1* changes have been described in patients with infantile lactic acidosis, deafness, and early death^[168]^. Variants in the *MRRP2* subunit induce developmental regression, seizures, and involuntary movements^[169]^. The remaining mt-tRNA is then released fully by cleavage at the 3’ end by RNAase Z (*ELAC2*). This cleavage releases the mt-tRNA from the mt-mRNA and mt-rRNA within the long polycistronic transcript. Pathological variants in *ELAC2* have been shown to give rise to infantile hypertrophic cardiomyopathy, global developmental delay, and early death^[170]^. Rare patients have expressed abnormal involuntary movements, acanthocytosis, and psychosis and live into adulthood^[171]^. PNPase, encoded by *PNPT1*, is a critical enzyme in polycistronic mtRNA transcript metabolism and likely import^[172]^. The range of disease is wide with some patients expressing sensorineural hearing loss, choreoathetosis, visual loss, and cataracts. Some patients express Leigh syndrome.

Once the mt-tRNA has been excised from the polycistronic mRNA, the released 11 mtDNA-encoded subunits are clipped. The ND6 mt-mRNA is immediately ready for translation without further processing. The other subunit mt-mRNAs are polyadenylated performed by mtPAP, a mitochondrial polyA polymerase encoded by *MTPAP*. Dysfunctional mtPAP produces range of disease, with a more common presentation of a progressive spastic ataxia, optic atrophy, and learning difficulties, but some variants also induce an early infantile death^[173,174]^. The leucine-rich pentatricopeptide repeat-containing protein (LRPPRC) is found mainly in the matrix, where it controls mRNA stability^[175]^. Variants in the *LRPPRC* gene can produce the French-Canadian type of Leigh syndrome^[176]^. The fas-activated serine-threonine kinase (FASTK) family of proteins are RNA-binding proteins. One of these proteins, fas-activated serine-threonine kinase domain 2 (FASTKD2) is tissue specific, and variants in *FASTKD2* have been linked to developmental delay and myopathy^[177]^. In some tissues, FASTKD2 is responsible for ND6 messenger RNA and 16S ribosomal RNA stability^[178]^. There are three proteins responsible for maturation of the 16S mt-ribosome: MRM1, MRM2, and RNMTL1. These proteins are responsible for the 2’-O-ribose modification. A MELAS-like syndrome has been reported with *MRM2* variants^[179]^.

The isolated mt-tRNAs need further modifications, likely due to their poor stability. The inherent instability of the mt-tRNA creates a hot-spot for disease-causing changes. Indeed, 45 pathological variants and over 200 variants are thought to be disease related (http://www.mitomap.org/MITOMAP). Two types of modifications occur in mt-tRNA, structure and codon-anticodon recognition. The order of mt-tRNA modification is not completely clear. Methylation is critical for obtaining proper cloverleaf structure of the mt-tRNAs. Early modification of methylation occurs during the 5’ processing and before cleavage at the 3’ end, with the RNase P methyltransferase activity of MRRP1 (also known as TRMT10C) and the gene product of *HSD17B10*, MRRP2^[180]^. Codon and anticodon recognition occur after 3’ cleavage. There are several mt-tRNAs that require modifications to stabilize the U-G wobble pairing. Precursor tRNAs are modified at the 3’ end by tRNA nucleotydyl transferase 1, which adds a CCA sequence^[155]^. The methyltransferase TRMT5 modifies tRNA at position G37 to contribute to the high fidelity of codon recognition in several mt-tRNAs^[181]^. The exact mechanism remains unclear, but taurine medication is required for five mt-RNAs, tRNA^Glu^, tRNA^Trp^, tRNA^Lys^, tRNA^Leu(UUR)^, and tRNA^Gln^. The gene products from *MTO1* and *GTPBP3* are intimately responsible for the 5-taurinomethyl group addition of these tRNAs while TRMU catalyzes the thiolation of tRNA^Glu^, tRNA^Gln^, and tRNA^Lys[182]^. Clinically, patients with pathological variants in mt-tRNAs and processing and modification enzymes have expressed liver failure, cardiomyopathy, lactic acidosis, and, for unclear reasons, a reversible liver disease [Table 3]^[181–183]^. Other patients have been found to have hypertension, myoclonic seizures, deafness, and spastic paraparesis.

Aminoacyl-tRNA synthetases (ARS) are specific enzymes involved in translation. There are 20 mt-tRNAs, and each has to be charged by one of the 19 specific ARSs. These enzymes catalyze a two-step reaction, where the ARS activates the amino acid with ATP to form an aminoacyl-adenylate and then transfers the aminoacyl group to the bound tRNA^[184]^. There are 17 nuclear-encoded ARSs that are specific for mt-tRNA and 2 (Glycyl- and Lysyl-tRNA synthetases) that are shared with the cytoplasm. One specific mt-tRNA synthetase complex, GatCAB amino-tRNA amidotransferase complex, has been identified; GLN-tRNA^Gln^ is synthesized indirectly via misacylation via transamidation^[185]^. The GatCAB complex consists of gene products of *QRSL1*, *GATB*, and *GATC* required for aminoacylation and subsequent protein translation^[186]^. Pathological variants in each of these GatCAB subunit genes have been found to induce a severe cardiomyopathy. Translation begins with N-formyltransferase (MTFMT) using the substrate mettRNA^Met^ and 10-formyl-tetrahydrofolate. Subsequently, binding of a methyl group to the wobble position of mt-tRNA^Met^ by *NSUN3* to enhance base pairing^[187]^. Patients with variants in *MTFMT* and *NSUN3* have been described with microcephaly, developmental delay muscular weakness, and CPEO. All mitochondrial ARS have been associated with autosomal recessive disease. Disease manifestations range from single organ to multisystem dysfunction, including Leigh syndrome^[39,184]^. Single organ disease has been noted to be isolated to the central nervous system (CNS) with a leukoencephalopathy and lesions in certain neuronal cell types, and in isolated peripheral neuropathies, distal myopathy, and renal tubulopathy. Multisystem disease is also noted, including a Leigh syndrome phenotype, Alpers-Huttenlocher syndrome, Perrault syndrome, myopathy, lactic acidosis, sideroblastic anemia, spastic paresis, atypical Charcot-Marie-Tooth disease, loss of cognitive ability, ataxia, and endocrinopathies. Why there is isolated single organ involvement versus multisystemic disease remains unclear.

#### Mitochondrial RNA translation

The initiation of the translation process begins by the recruitment of the mt-mRNA to the small mt-rRNA subunit. The initiation factor, mtIRF, then promotes the dissociation of the mitoribosome into two subunits and prevents premature reassociation with the larger mt-rRNA subunit^[188]^. Subsequently, the charged fMET-tRNA binds at the P site of the small mt-rRNA subunit. With the alignment of the start codon triplet bound to the anti-codon triplet, stabilization the complex occurs and subsequent association of the larger mt-rRNA subunit is induced. Once this monoribosome is formed, elongation begins. Variants of the modifiers of translations, elongation, termination, and protein release factors can cause disease. Three of the many elongation factors, EFTu, EFTs, and EFG1 (products of the nuclear-encoded genes *TUFM*, *TSFM*, and *GRM1*), are involved in disease. Pathological variants in each of the three genes induced hepatopathy and encephalopathy^[39,189,190]^. The protein products of *GRM2* and *IFG2* function at the termination step of translation to disassemble the mitoribosome and allow subsequent cycles of protein synthesis^[191]^. The gene *C12orf65* is a member of the mitochondrial release factor family, the exact properties of the gene product remain unclear, it but has been shown to cause a diverse phenotype with the key features of optic atrophy, peripheral neuropathy, and spastic paraparesis^[192]^.

The mechanisms to enhance translation mt-mRNA are relatively unknown. The translational activator of cytochrome oxidase subunit 1 (TACO 1) specifically binds mt-Co1 mRNA and is required for translation of COX1 with association with the ribose^[193]^. Pathological variants induce Leigh syndrome, optic atrophy, and dystonia.

### Mitochondrial protein import and processing

#### Import

There are only 13 proteins produced by the mitochondrial genome. With the estimated 1500 proteins needed for organelle structure and function, specialized protein import systems have evolved to get specific mitochondrial proteins to their sub-organelle location. A full description of the import systems is beyond the scope of this paper but was recently reviewed by Pfanner *et al*.^[194]^. Briefly, approximately 60% of the nuclear-encoded proteins possess specific targeting signals that direct gene products from the cytosol to the mitochondrial surface receptors and subsequently into mitochondrial subcompartments. Precursor proteins are synthesized with a targeting N-terminal positively charged presequence. These proteins pass from specific OMM translocase of the outer membrane (TOM) complex to a presequence translocase of the inner membrane (TIM) complexes, TIM23 and TIM22. The protein then passes into the matrix by the presequence translocase-associated motor. Nontargeted containing proteins use the TOM channel for translocation, although the mode of delivery is likely different and depends on internal targeting signals. There are small TIM chaperones of the intermembrane space that guide these complexes to the translocated of the inner membrane (TIM22 complex). Many of these proteins contain cysteine motifs that are needed for translocase.

Pathological variants in *acylglycerol kinase* (*AGK*) induce Senger syndrome, which is a rare recessive disorder characterized by lactic acidosis, hypertrophic cardiomyopathy, and bilateral cataracts. AGK is a component of the TIM22 complex and is essential for import and assembly of metabolite carrier proteins in a kinase-independent manner^[195]^. A component of the TIM23 complex, TIMM50, is essential for importing proteins into the inner compartment^[196]^. The transporter TIMM8A is part of a complex of TIM proteins that facilitate the import of proteins across the inner membrane space by acting as a chaperone to keep the hypdrophobic substrate unfolded^[197,198]^. *TIMM8A* is an X-linked gene that is associated with deafness, dystonia, optic neuronopathy, and Mohr-Tranebjaerg syndrome. The X-linked *AIFM1* gene encodes a multifunctional protein, a mitochondrial apoptosis-inducing factor, which is a FAD-containing and NADH-specific oxidoreductase. The AIFM1 protein is important for energy metabolism and involved in the caspase-independent cell death pathway. In the inter-mitochondrial space, it contributes to folding of some of the ETC subunits. Depending on genetic changes, *AFIM1*-induced disease can range from infantile onset of severe neurodegeneration to a slowly progressive disorder^[199]^. The gene product of *DNAJC19* is complexed with another set of proteins, prohibitins, that form protein and lipid scaffolds in the inner mitochondrial membrane. DNAJC19 complex is also involved in protein translocation^[200]^. The exact role of DNAJC19 is not fully characterized in humans, but pathological variants have been found to induce a phenotype of cardiomyopathy and ataxia, similar to Barth syndrome^[201]^. The *GFER*-encoded protein is essential for disulfide protein folding in the intermitochondrial space. Variants induce an infantile onset congenital cataract, sensorineural hearing loss, and developmental delay with multiple mtDNA deletions^[202]^. *MIPEP* encodes a peptidase that is required for secondary processing within the matrix. Variants in this gene has been found in patients with cardiomyopathy, developmental delay, seizures, and early death^[203]^. *PMPCA* encodes the alpha subunit of the mitochondrial precursor peptidase, the primary enzyme responsible for the maturation of most of the nuclear-encoded proteins entering into the matrix^[204]^. Phenotypically, patients with variants have a non-progressive cerebellar ataxia.

#### Processing

As would be expected in a highly coordinated quality control system involving transport across two membranes and intramembrane space, errors are common. In addition, proteins are modified within the matrix compartment for functionality. The mitochondrial protease system has evolved to prune abnormal proteins and preserve functional integrity. There are three different classes, namely cysteine proteases, metalloproteases, and serine proteases: whose functions are to remove import signals, degradation of misfolded and damaged proteins, and determine half-life of short-lived regulatory proteins^[205]^. There are at least 10 diseases associated with loss of protease function: seven are recessive (*CLPP*, *LONP1*, *PARK7*, *PARL*, *SPG7*, *UQCRC2*, and *XPNPEP3*), two are dominant (*HTRA2* and *IMPP2L*) and one has both dominant and recessive modes of inheritance (*AFG3L2*).

Nonketotic hyperglycinemia (NKH) is an autosomal disorder characterized by variants in the glycine cleavage system and lipoate synthase^[206,207]^. NKH due to variants in the lipoate synthetase are described in the next section below on Iron Cluster Biosynthesis and Mitochondrial Iron Homeostasis. The glycine cleavage system is composed of three enzymes, P, T, and L subunits, and one carrier protein, H subunit^[208]^. The majority of disease-causing variants are in the *pyridoxal phosphate-dependent glycine decarboxylase* encoded P subunit with the remaining in the *amino methyltransferase* encoded T subunit.

The process of fusion and fission, also named mitochondrial dynamics, in part monitors healthy and impaired mitochondria in response to physiological signals and metabolic stress. Proteases play a role in these processes. Damaged mitochondria undergo degradation by mitophagy^[209]^. PINK1 and PARKIN are two proteins that mediate this autophagy process. PINK recruits PARKIN to damaged mitochondria and the Presenilin Associated Rhomboid-Like (PARL) protease within the inner mitochondrial membrane and induces mitophagy^[210]^. Rare recessive pathological variants in *PARL* results in PINK1 not being cleaved during protein damage and results in accumulation of abnormal proteins with subsequent incomplete mitophagy. The cascade of altered PARL function is associated with induction of Parkinson disease^[211]^. XPNPEP3 is a protease thought to be involved in post-translation modifications of proteins controlling their half-life^[212]^. Recessive pathological variants in *XPNPEP3* give rise to a nephronophthisis-like nephropathy^[213]^. Multiple etiologies of hereditary spastic paraplegia (SPG) exist. SPG type 7 is due to alterations in paraplegin generating gene, *SPG7*^[214]^. Other phenotypes of *SPG7* variants induce CPEO, optic atrophy, and muscle showing multiple mtDNA deletions^[215]^. Alterations in other essential proteases can cause disease. Variants in *PMPCB*, which encodes the catalytic subunit of mitochondrial processing protease, induce a progressive neurological disease in early childhood^[216]^. LonP1 is one of the members of the highly conserved AAA+ superfamily of soluble proteases found in the matrix that selectively processes a variety of proteins to both activate and degrade damage proteins. Recessive variants in the *LONP1* gene have been found to cause Leigh syndrome and the syndrome of cerebral, ocular, dental, auricular, and skeletal anomalies^[217,218]^. Another of the matrix proteases, IMMP2L, is associated with removal of the signal N-terminal peptide within proteins. Variants in *IMMP2L* have been associated with a small subset of patients with Tourette syndrome with dominant inheritance^[219]^. The mitochondrial ATP-independent serine protease HTRA2 resides in the intermembrane space and provides protein homeostasis, as well as is involved in apoptosis. Recessive and dominant variants in *HTRA2* induce disease ranging from infants to adults. Adult onset has been associated with dominant *HTRA2* variants inducing tremor, while recessive variants cause adult onset Parkinson’s disease and infants with recessively inherited seizures, dysphagia, hypotonia, apnea, and cataracts^[220,221]^. CLPP is an ATP-dependent matrix protease and is also involved in ribosome assembly within the inner membrane. Variants in *CLPP* induce Perrault syndrome (type 3), which presents with sensorineural hearing loss and ovarian failure^[222]^. Several of the AAA proteases lie within the inner membrane. When the catalyst domain lies facing the inner membrane space, it is given the name of i-AAA, and when facing in the matrix m-AAA protease. The i-AAA protease YME1L is involved with dynamics and metabolic integrity of the organelle^[223]^. Variants in *YME1L* alter OPA1 processing, which induces imbalance between fission and fusion with the phenotype of optic atrophy^[224]^. Another member of the i-AAA proteases, AFG3L2, assembles into oligomeric isoenzymes or with SPG7 subunits. Variants in *AFG3L2* produce dominant spinocerebellar ataxia and recessively inherited spastic-neuropathy syndrome (SCA28), while *SPG7* induces recessive inherited spastic paraplegia^[225]^. The m-AAA ATPase, ATAD3A, lies in the IMM with contact sites with the OMM. The *N*-terminal domain interacts with the inner surface of the OMM with the C-terminus ATPase domain in the matrix^[226]^. Alterations in *ATAC3A* are involved in fission, mitochondrial fragmentation, cholesterol synthesis (a component of mtDNA nucleoid maintenance), and protein translation. Dominant variants in this gene express global developmental delay, hypotonia, optic atrophy, axonal neuropathy, and cardiomyopathy and isolated hereditary spastic paraplegia^[227,228]^.

### Iron-sulfur cluster biosynthesis and mitochondrial iron homeostasis

A strict balance of iron (Fe) and sulfide ion (S) concentrations is maintained to prevent their damaging oxidative properties when present in excess. Complex biosynthetic systems have evolved to “protect” the cell, while incorporating Fe/S clusters into specific apoproteins for cellular functioning. To date, there are at least 18 known Fe/S cluster assembly proteins involved in proper biogenesis and trafficking of Fe/S containing clusters within mitochondria. A full review of this process is beyond the scope of this article but can be found elsewhere^[229–231]^.

The incorporation of Fe/S clusters into mitochondrial proteins involves initial assembly into 2Fe/2S complexes within the mitochondrion on a specific scaffold protein complex. Fe and S are complexed together by specific transfer proteins into the 2Fe/2S complex, subsequently converted into the 4Fe/4S and 3Fe/4S complexes, and then inserted into Complexes I, II, and III. The 2Fe/2S clusters are trafficked to the late-acting machinery for 4Fe/4S cluster synthesis. During the late synthetic process, there is one 3Fe/4S cluster that is inserted into Complex II. Once fully synthesized, the 4Fe/4S clusters are inserted into the ETC Complexes I and II. Complex III only contains a single 2Fe/2S complex.

The assembly of iron and sulfur complexes is a highly regulated process and the mechanisms involved are not completely understood. Intracellular iron homeostasis is controlled post-transcriptionally by iron-regulatory protein 1 (IRP1) and 2 (IRP2). The IRPs bind to RNA stem-loops known as iron-responsive elements of mRNAs involved in iron uptake and sequestration, transferrin receptor expression, and ferritin levels. IRP2 is intimately involved in iron homeostasis via an oxygen-responsive iron-sulfur cluster. Although both IRPs are members of the aconitase family of proteins, IRP2 cannot form a 4Fe/4S cluster and does not express aconitase function. Mouse models of IRP2 deficiency leads to reduced transferrin receptor expression and reduced iron acquisition^[232]^. The connection to mitochondrial function comes from mouse models and rare patients with pathological variants, which confirm a mitochondrial dysfunction^[232,233]^.

Ferredoxins are iron-sulfur proteins involved in the early cluster assembly. Ferredoxin reductase (FDXR) donates electrons from NADPH to homologous ferredoxins, FDX1 and FDX2, and initiates the 2Fe/2S scaffold complex. Variants in *FDXR* diminished iron-sulfur cluster assembly and induce mitochondrial iron overload^[234]^. The diminished cluster assembly phenotypically gives rise to optic atrophy and sensory neuropathy^[235,236]^. Recessive mutations in *FDX2* induces a complex phenotype consisting of optic atrophy, reversible leukoencephalopathy, myopathy, and axonal polyneuropathy^[233,236]^. The scaffold protein iron-sulfur cluster assembly enzyme ISCU serves as the initial step of the 2Fe/2S complex formation. Sulfur, arising from cysteine, is shuttled to the ISCU by the cysteine desulfurase, NFS1. The ISCU complex consists of at least the NFS1, ISD11, ISCU, and Frataxin (FXN) proteins^[229,230]^. Recessive pathological variants have been described in all of the early scaffold assembly complex genes. A recessive infantile onset disorder with ETC Complex II and III deficiency, multisystem organ failure, and hypotonia has been described with variants in *NFS1*^[237]^. *LYRM4* encodes the ISD11 protein, which forms a complex with, as well as stabilizes, the sulfur donor NFS1. Recessive mutations in the *LYRM4* induces deficiency of Complexes I-III in muscle and liver^[238]^. Another component of the initial iron-sulfur scaffold, variants in *ISCU* induce a myopathy and exercise intolerance. Recessive variants in *ISCU* in muscle have been demonstrated to induce myopathy^[239]^. Friedreich ataxia, the most common autosomal recessive ataxia, is due to homozygous expansion of a GAA trinucleotide repeat in intron 1 of *FXN*^[240]^. Deficiency in the *FXN* encoded FXN protein leads to a progressive spinocerebellar neurodegeneration associated with gait and limb ataxia, dysarthria, muscle weakness, cardiomyopathy, and diabetes.

The second step, newly synthesized 2Fe/2S cluster, is trafficked to the late-acting iron-sulfur machinery for 4Fe/4S cluster synthesis and mitochondrial export. How the Hsp70 chaperone system shuttles the 2Fe/2S complex from early to the late acting iron-sulfur synthesis complex is not well understood in higher order eukaryotes. The chaperone complex, consisting of the proteins GRP75, HSPA9, HSCB, GRPEL1/2, and GLRX5, extracts the 2Fe/2S complex and transfers the complex to glutaredoxin Grx5. Recessive mutations in *GLRX5* induce nonketotic hyperglycinemia with neurodegeneration, leukoencephalopathy, optic atrophy, and spastic paraplegia in childhood^[206]^.

The final maturation of the 2Fe/2S complex into the 4Fe/4S complex occurs in the complex containing the mammalian A-type proteins ISCA1 and ISCA2, and the folate binding protein IBA57^[49]^. Due to the downstream delivery proteins, deficiency of any constituent of this maturation or late-acting complex results in loss of functional ETC and lipoic acid. Delivery proteins, Nfu1, BOL1, and BOLA3, are then used to specifically target 4Fe/4S clusters to the proper apoprotein. Recessive variants in the genes of the late maturation of 4Fe/4S complex and dysfunctional delivery proteins give rise to the group of multiple mitochondrial dysfunction syndromes (MMDS). There are five different MMDS encoded by *NFU1* (MMDS1), *BOLA3* (MMDS2), *IBA57* (MMDS3), *ISCA2* (MMDS4), and *ISCA1* (MMDS5). Clinically, these diseases are characterized by infantile encephalopathy, non-ketotic hyperglycemia, myopathy, early death, and leukoencephalopathy^[206–208,241]^. In animals, models of cardiac hypertrophy are produced by *ABCB7* transporter variants, an iron transport membrane protein that alters Complex IV and V function and contributes to heart failure^[242]^. However, human studies or patients have not been described.

Neurodegeneration with brain iron accumulation (NBIA) are a group of neurodegenerative diseases characterized by progressive central nervous system dysfunction and iron accumulation. To date, there are 10 candidate genes that have been identified with four located within mitochondria, *PANK2*, *COASY*, *PLA2G6*, and *C190rf12*. Pantothenate kinase 2 encoded by *PANK2* is located within the intermembrane space and phosphorylates vitamin B5 in the first reaction of the CoA biosynthetic pathway. Iron accumulation occurs in the globus pallidus, substantia nigra pars reticularis, cerebellar white matter, nucleus gracilis, and dentate nucleus^[243]^. Disease onset is usually young childhood and presents with a progressive dystonia, rigidity, dysarthria, and spasticity. *C19orf12* encodes a mitochondrial membrane protein of unknown function. Variants in *C19orf12* induce a progressive spastic paraplegia, optic atrophy, motor axonal neuropathy, and psychiatric problems and has been named mitochondrial membrane protein-associated neurodegeneration^[244,245]^. Age of onset can vary from childhood to well into adulthood, and both autosomal recessive and dominant types have been described^[244,245]^. Variants in *COASY* lead to an early-onset autosomal recessive form of NIBA, which has been named COASY protein-associated neurodegeneration. The COASY protein is found mostly in the matrix, but also has been identified on the outer membrane and catalyzes the final steps of CoA biosynthesis^[246]^. Variants induce a mild oromandibular dystonia with dysarthria, spastic-dystonic gait, severe Parkinsonism, areflexia, and eventual loss of gait. The solute carrier, SLC25A42 is thought to transfer CoA into the mitochondrial matrix. Recently variants in *SLC24A42* have been associated with developmental delay, epilepsy, dystonia, and basal ganglia lesions^[247]^. Another iron related protein, iPLA2, is encoded by *PLA2G6* and is a calcium-independent phospholipase A2 group VI that releases free fatty acids and lysophospholipids from glycerophospholipids^[248]^. iPLA2 is localized to the mitochondria and cytoplasm. Loss of iPLA2 activity has been associated with increased mitochondrial lipid peroxidation, loss of membrane potential, and decreased ATP synthesis and abnormal organelle morphology^[249]^. Recessive mutations cause three different but overlapping phenotypes: classic infantile neuroaxonal dystrophy, atypical neuroaxonal dystrophy, and *PLA2G6*-related dystonia-Parkinsonism. The exact mechanism of mitochondrial involvement is not completely understood. Phenotypically, mutations cause a cerebellar ataxia, psychomotor regression, neuroaxonal dystrophy, dystonia, and Parkinsonism^[250]^.

Lipoic acid synthase (LIAS) is a 4Fe/4S containing enzyme and interacts with S-adenosylmethione to donate sulfur for lipoic acid formation^[251]^. LIAS intersects fatty acid synthesis at the formation of octanoylacyl-carrier protein and three lipoate-specific steps. Octanoic acid is transferred to the glycine cleavage H protein by LIPT2 and lipoate transfer protein LIPT1, which is necessary to donate lipoylate to the E2 subunits of the 2-oxoacid dehydrogenases. Recessive variants in *LIAS* result in nonketotic hyperglycinemia with early onset seizures, cardiomyopathy, and encephalopathy^[206–208]^. Lipoic acid is a required cofactor for multiple enzyme functions in the mitochondria: pyruvate dehydrogenase, alpha ketoglutarate dehydrogenase, 2-oxoadipate dehydrogenase, branch-chain ketoacid dehydrogenase, and glycine cleavage system. Recessive variants in *LIPT1* and *LIPT2* can induce a variety of clinical range of disorders from Leigh syndrome to non-ketotic hyperglycemia, hypotonia, seizures, microcephaly, psychomotor retardation, and cardiomyopathy^[251]^.

SFXN4 is one of the sideroflexin proteins, with an unknown function in mitochondria. There is data to suggest that SFXN4 is involved in Fe-S cluster biogenesis, as knockout cells decrease Fe-S-containing proteins, regulate IRP1 and IRP2 expression and decreased ETC activity^[252]^. In another study, SFXN4 was localized to the inner mitochondrial membrane^[253]^. Variants in *SFXN4* induce intrauterine growth retardation, microcephaly, vision impairment, and macrocytic anemia^[253]^.

### Mitochondrial dynamics: membrane transport and fusion/fission dynamics

The cell biology of mitochondrial dynamics is expanding our understanding of intracellular signaling coupled to cellular mitochondrial networks. Mitochondrial dynamics orchestrate metabolism, regulate cell pluripotency, cell division, differentiation, senescence, and cell death. The complete description is beyond the scope of this article, but excellent reviews exist^[41,254,255]^. Briefly, various physiological functions are intimately related to mitochondrial morphology as driven by fusion and fission. Fission involves the splitting a mitochondrion into smaller, more discrete mitochondria. Depending on the cellular context, fission can accelerate cell proliferation, generate oxygen radical species, or facilitate mitophagy. Fusion creates a more interconnected mitochondrial network that enhances communication with the ER. Fusion also allows diluting the accumulated mtDNA mutations and oxidized proteins. Fission and fusion are mediated by a number of guanosine triphosphatases (GTPases) and small outer membrane partners: mitochondrial fission factor (MFF), mitochondrial fission 1 protein (FIS1), mitochondrial elongation factor, mitofusion-1 (Mfn-1) and −2 (Mfn-2), and inner membrane protein (OPA1). There are also small vesicles excised from mitochondria, mitochondrial derived vesicles (MDVs), which contain OMM and IMM proteins that can fuse with other organelles. The exact role of MDVs has not been fully elucidated but they are thought to be communication vesicles to other organelles. Their formation is not related to GTPase or dynamin function. Other budding vesicles, referred to as mitochondrial-derived compartments (MDCs), also carry outer and IMM proteins. These vesicles are dynamin-mediated fission products and are thought to be involved in quality control. Mitochondria are also involved in apoptosis in association with BCL-2, which controls the mitochondrial OMM permeabilization and subsequent fragmentation with cytochrome C release. The OMM has mitochondrial antiviral signaling proteins (MAVS) that are involved in innate immunity and signal transduction.

There are several functions involved in these dynamic processes. Mitochondrial biogenesis is regulated by cellular energy demands and compensation for cell damage. This proliferation and pruning process is mediated by the peroxisome proliferator-activate receptor γ coactivator 1a (PCG-1*α*) with the fusion mediator Mfn-2. Fission and fusion dynamics are involved in a quality control process named mitophagy. This autophagy process maintains cellular health by isolating depolarized and hence dysfunctional mitochondria by fission, while coordinating downregulation of fusion mediators preventing persistence of damaged mitochondria by active degradation. Mitochondria are linked to the ER at specialized regions known as mitochondria-associated ER membranes by protein bridges (MERCs). A key tethering protein is MFN-2 as well as another linking protein from the ER named ER-resident vesicle-associated membrane protein-associated protein V (VAPB) with another outer membrane protein, tyrosine phosphatase-interacting protein 51 (PTPIP51). The interaction of mitochondria at these specialized ER membrane sites are involved in calcium flux into mitochondria and important for calcium homeostasis and metabolism. Calcium imbalance may initiate mitophagy or at physiological concentrations enhance oxidative phosphorylation. In addition to mitochondria being associated with the ER at specific positions, mitochondrial also traverse the cytosol and axons on dynein and kinesin tracks.

Fusion and fission are key processes regulating normal mitochondrial function and preventing disease^[255]^. Fission is performed by the dynamin-related protein 1 (DMN1L, also known as DRP1). This protein translocates from the cytosol to the mitochondria outer membrane and binds to mitochondrial fission factor (MFF), mitochondrial dynamics protein of 49 kDA (MID49), and fission 1 protein (FIS1). The binding of DMN1L creates a band around the mitochondria and induces fission. Disruption of fission results in longer, abnormally distributed mitochondria and peroxisomes. Pathological variants in *DNM1L* induce a disorder known as Encephalopathy due to Defective Mitochondrial and Peroxisomal Fission 1 first described in 2007 in a newborn with microcephaly and optic atrophy^[256]^. Over time, other variants expanded the phenotypes with recessive and dominant variants. Isolated optic atrophy and others with seizures, microcephaly, and global developmental delay were noted in both recessive and dominant inheritance patterns^[257]^. Rare recessive variants in *MFF* have been reported to give rise to epilepsy, encephalopathy, hypotonia, and Leigh syndrome^[258]^. The ganglioside-induced differentiation-associated protein 1 (GDAP1) is located within the outer mitochondrial membrane. This protein promotes mitochondrial fission through interactions with DMN1L and FIS1^[259]^. Variants in GDAP1 produce both dominant and recessive forms of Charcot-Marie-Tooth disease (CMT), a group of motor or sensory neuropathy diseases. The recessive forms have reduced fission activity and phenotypically have earlier onset with a more severe demyelinating subtype of CMT (CMT4A) or a later onset intermediate axonal form that is less involved (ICMT). In dominant disease, variants in *GDAP1* interfere with fusion and induce a much milder CMT disease (CMT2K). Mfn-1 and −2 are OMM dynamin-like GTPase proteins involved in fusion^[260]^. Mfn-2 is involved in tethering mitochondria with ER membranes. Recessive variants in *Mfn-2* are the most common cause of axonal induced CMT 2A and produce mtDNA deletions and depletion^[261]^.

Optic atrophy 1 (OPA1), a mitocholndrial dynamin-like GTPase, is anchored to the IMM within the intermembrane space and plays a critical role in fusion and mtDNA maintenance. OPA1 anchors mtDNA to the IMM, organizes ETC supercomplexes and cristae structure, regulates Ca2+ homeostasis, and complexes with soluble cytochrome c within the cristae^[262]^. Depending on location and type of pathological variant within OPA1, multiple types of disease manifestation occur. The classic autosomal dominant form of optic atrophy is due to nonsense or frameshift variants (haploinsufficiency), while missense variants within the GTPase domain produce a dominant negative effect and multisystem disease^[263]^. Phenotypically, patients express optic atrophy and retinal ganglion cell layer loss in the classic dominant form, while “plus” patients, missense variants, display optic neuropathy, sensorineural deafness, ataxia, CPEO, myopathy, and sensorimotor polyneuropathy. The fusion protein MSTO1 is localized mainly to the OMM. *MSTO1* recessive and dominant variants induce abnormalities in mitochondrial fusion and aggregation of mitochondria at the perinuclear region^[264]^. Dominant *MSTO1* variants have been found in patients with myopathy, ataxia, optic atrophy, and developmental delay, while recessive variants induce myopathy and cerebellar ataxia^[265,266]^. Cytoplasm localization of MSTO1 in some studies suggest the exact location to be unclear. Sacsin, encoded by *SACS* is a protein that is thought to disrupt DMN1L function and alter the balance between fusion and fission^[267]^. Recessive variants in *SACS* induces a childhood onset spastic ataxia disorder, autosomal recessive spastic ataxia of Charevoix-Saguenay. Once thought to be limited to patients living in Quebec, further findings have identified patients in over 13 countries worldwide. Trak1 is required for mitofusin-mediated fusion and is localized and interacts with Mfn1 and Mfn2 on the OMM. Trak1 can also interact with Mfn1 and Mfn2 to act as a tethering factor in fusion^[268]^. Recessive variants have a range of disorders from isolated hypertonia to patients having fatal encephalopathy, seizures, delayed myelination, hyperplexia, and refractory status epilepticus^[269,270]^.

SLC25A46 belongs to the mitochondrial transporter family but is not known to have transport function. The protein is located on the OMM and whose function is not clearly defined, but interactions with OPA1, Mfn-1, and Mfn-2 suggests a function in fusion and maintenance of cristae junctions^[271]^. Variants in *SLC25A46* produce a wide spectrum of clinical features, with optic atrophy and axonal neuropathy shared by most all patients. Early death has been reported, with Leigh syndrome or ponto-cerebellar hypoplasia. Other patients present later in life with optic atrophy diffuse brain and cerebellar atrophy^[272,273]^.

STAT (signal transduction and activation of transcription) proteins are regulators of early response genes in the nucleus and roles in mitochondrial functions are beginning to be described. One of the STAT proteins, STAT2, protects cells from viruses by an interferon-dependent mechanism. In response to a viral infection, STAT2 translocates to the mitochondria, and likely attenuates the cell’s anti-viral response and suppresses innate immunity^[274]^. Two patients with homozygous variants within *STAT2* were found to have significantly decreased phosphorylated serine at serine 637 of DMNL1, with impaired fission in the context of receiving measles, mumps, and rubella vaccination^[275]^. Patients were healthy until vaccination, but both soon afterward developed lethargy and lymphadenopathy. Subsequently, one patient developed episodes of opsoclonus-myoclonus, intractable seizures, and visual impairment. The other developed septic shock but recovered. The variants in *STAT2* are likely ecovariants that lie silent until exposed to specific environmental stimuli (see above discussion).

The Yeast Vacuolar Protein Sorting-associated Protein (VPS13D) acts downstream of the recruitment of the fission factor DMN1L to control fission and clearance by mitophagy^[276]^. Recessive variants have been shown to induce a range of diseases from spastic ataxia, chorea, and dystonia to isolated spinocerebellar ataxia, ranging in onset from infancy to the third decade of life^[277]^. Two other members of this protein family, VPS13A and VPS13C, are thought to help tether mitochondria to the ER and act as a lipid transporter. Recessive variants in *VPS13A* induce chorea acanthocytosis and in *VPS13C* early onset Parkinson disease^[278]^.

The F-box and Leucine rich repeat protein 4 (FBXL4) is located in the intermembrane space and recessive *FBXL4* variants have been shown to induce severe mtDNA depletion with infant onset encephalopathy^[279,280]^. Recently, fibroblast cultures derived from patients demonstrated reduced fusion rates, but genetically engineered cells with overexpression of FBXL4 were found to express mitochondrial hyperfusion, suggesting FBXL4 involvement in fusion^[280]^. An unanswered question of how variants induce mtDNA depletion remains. Phenotype of biallelic variants consists of early onset, malformation of cortical development, kidney disease, encephalopathy, skeletal abnormalities, and early death^[279–281]^.

Extensive mitochondrial damage promotes release of pro-apoptotic factors from the intermembrane space. Release of the proteins inhibit the cytosolic E3 ubiquitin ligase XIAP and other inhibitors of apoptosis proteins, one of these proteins is HTRA2/Omi. Recessive variants in *HTRA2* induce hypotonia, extrapyramidal symptoms, lack of psychomotor development, and seizures^[282]^.

The optic atrophy 3 (OPA3) protein has been localized to the IMM in animal studies and OMM in human cell culture. Mitochondrial fragmentation found in patient fibroblasts suggests OPA3 is involved in fusion, but the exact mechanism remains unclear^[283]^. Loss-of-function recessive and dominant-negative variants have been described, with phenotypic overlap. Missense biallelic *OPA3* variants were first described in an Iraqi Jewish community to have infantile optic atrophy, movement disorder, spastic paraparesis, ataxia, seizures, and cognitive impairment, now known as Costeff syndrome^[284]^. Other ethnic groups also have been reported. Dominant *OPA3* variants demonstrate optic atrophy, cataracts, lipodystrophy, seizures, and peripheral and autonomic neuropathy^[285]^.

The TMEM65 protein is located in the IMM, and, in siRNA experiments on patient fibroblasts, it altered respiration rate and mitochondrial content^[286]^. The sole patient reported with biallelic *TMEM65* variants displayed microcephaly, seizures, vision loss, and global developmental delay^[287]^. The exact function of the TMEM65 protein remains unknown.

#### Mitochondrial membrane modification/homeostasis

Phospholipids are structural components of membranes, but also physiological active within multiple cellular processes, act as vesicles with intracellular signaling transduction and involved in mitochondrial fusion and fission. Mitochondria contain the unique phospholipid cardiolipin, which is predominantly found in the IMM with also a minor component of the OMM. The biophysical properties of cardiolipin make it a non-bilayer forming acidic phospholipid fitting into the inner side of the curved IMM, characteristic of the mitochondrial cristae. Cardiolipin is also involved in mitochondrial apoptosis and stabilizing of ETC supercomplexes. The major site of lipid synthesis is the ER and transported via contact points with mitochondria. Mitochondria are also intimately involved in beta-oxidation of fatty acids, unique fatty acid synthesis, lipid cofactors, and steroid hormone production.

The X-linked gene, *TAZ*, encodes an acyltransferase that catalyzes the remodeling of cardiolpin^[288]^. In cardiac and skeletal muscle, the predominant species of cardiolipin is reduced in favor of different acyl composition in *TAZ* pathological variants. Structurally, the variants produce enlarged mitochondria with bound glycogen, and cristae become stacked with circular arrays in heart and muscle. Phenotypically, Barth syndrome presents dilated cardiomyopathy, prolonged QT interval, proximal myopathy, delayed motor milestones, exercise intolerance, mild learning disabilities, delayed puberty, and neutropenia^[288]^.

Choline kinase beta initiated the de novo biosynthesis of phosphatidylcholine. Recessive mutations in *CHKB* induce an early onset psychomotor delay, muscle weakness, and hypotonia known as megaconial muscular dystrophy^[289]^. Skeletal muscle is found to have enlarged mitochondria with alteration of IMM potential^[290]^.

SERAC1 is located at the contact sites between the ER and mitochondria associated membrane and involved in the remodeling of phosphidylglycerol. Recessive mutations in *SERAC1* induce 3-methylglutaconic aciduria, deafness, encephalopathy, and neuroimaging evidence of Leigh-like disease (MEGDEL) syndrome. Disease onset is early infancy with neonatal hypoglycemia, hypotonia, hepatitis, dystonia of hands and feet, and severe motor delay^[291]^.

The calcium-independent phospholipases are named group VI iPLA2s. The iPLA2s or patatin-like phospholipases (PNPLAs) contain lipase and nucleotide-binding consensus sequences that function to hydrolyze a free fatty acid and a lysolipid from membrane phospholipids. iPLA2 γ is encoded by *PNPLA8*, which is the predominant phospholipase in mammalian mitochondria^[292,293]^. Recessive variants in *PNPLA8* give rise to microcephaly, spasticity, cerebellar and brainstem atrophy, seizures, and muscle weakness^[292,293]^.

The protein complement component 1 Q subcomponent-binding protein (C1QBP) is located primarily in the matrix, with roles in inflammation, mitochondrial ribosome biogenesis, and regulation of apoptosis. Although the precise involvement in mitochondrial function is unknown, *C1QBP* variants induce severely impaired protein synthesis^[294]^. Patients exhibit cardiomyopathy and multisystem involvement as infants, while those presenting later in life have a myopathy and CPEO^[295]^.

RTN4IP1 has a mitochondrial targeted leader sequence, co-localizes with ER at mitochondrial contact sites and is associated with the outer membrane; however, the exact function is unknown^[296]^. Biallelic recessive variants in *RTN4IP1* induce early onset optic atrophy with seizures and mild cognitive impairment.

#### Metabolite solute carriers

Mitochondrial solute carriers, solute carrier family 25 (SLC25), represent a group of 53 proteins required for transport ions, inorganic metals, vitamins, and substrates into mitochondria that are required for physiological functioning. All family members possess three specific homologous sequence repeated domains, each with two transmembrane segments that identify *SLC25* genes. Solute transporter proteins cycle substrates between the cytoplasmic of substrate-binding in the intermembrane space and matrix delivery and vice versa. Carrier proteins do not have a mitochondrial targeting presequence. They have an internal targeting signal recognized by the Hsp70-Hsp90 chaperones that allows passage through the OMM to the IMM. Solute transporter proteins are named SLC25A1-SLC25A53. Eighteen different SLC25A transporters have been reported to cause disease. A complete review of this subject is beyond the scope of this article and the reader can refer to a recent review to enlighten the scope of these processes^[297]^.

Vitamin B1, or thiamine, is a critical cofactor in multiple metabolic processes in the cytosol, mitochondria, and peroxisome. Free thiamine is transported into the cell using multiple transporters: two specific transporters, SLC19A2 (thiamine transporter-1) and SLC19A3 (thiamine transporter-2), with four nonspecific transporters, SLC19A1 (folate transporter), SLC44A4 (human TDP transporter), SLC22A1 (organic cation transporter 1), and SLC35F3. Once thiamine is transported into the cell, thiamine is converted into thiamine pyrophosphate (TPP) by thiamine phosphokinase (TPK1), which is the metabolically active form of thiamine. Active TPP is transported into the mitochondria by the specific carrier SLC25A19, where it acts as a cofactor of pyruvate dehydrogenase complex, oxoglutarate dehydrogenase complex, and branched chain 2-oxo acid dehydrogenase complex. Recessive variants can induce well-defined phenotypes; *SLC19A2* induces thiamine responsive megaloblastic anemia or Roger’s syndrome; *SLC19A3* produces biotin thiamine responsive basal ganglia disease and Leigh syndrome; *TPK1* causes Leigh syndrome; and *SLC25A19* causes Amish microcephaly and episodic encephalopathy with progressive polyneuropathy^[298–300]^. All three recessive variants express acute encephalopathy and basal ganglia changes, with other features of seizures, spasticity, ophthalmoplegia, and peripheral neuropathy. The importance of diagnosing *SLC25A19*, *SLC19A3*, and *TPK1* variants is that all three diseases respond to supplemental thiamine, limiting the disease.

The mitochondrial citrate carrier, SLC25A1, is responsible for the export of citrate to the cytoplasm needed for lipid, dolichol, ubiquinone, and sterol synthesis. Recessive mutations in *SLC25A1* leads to neonatal-onset encephalopathy, severe muscle weakness, seizures, respiratory compromise, and lack of psychomotor development^[301]^. The mitochondrial copper carrier, SLC25A3, is found as two isoforms: the A form is found in heart and muscle, while the B form is in all the other tissues. The two forms differ by 13 amino acids. Both isoforms also act as a phosphate carrier^[302]^. Recessive variants in the *SLC25A3-isoform A* present with muscle hypotonia and hypertrophic cardiomyopathy^[303]^. A single family has been shown to have biallelic variants, one in *isoform A* and the second in *isoform B*^[303]^. ANT is a solute carrier in the IMM and exchanges matrix ATP for cytosolic ADP. There are four tissue-specific isoforms of this carrier, ANT1–4, each encoded by separate genes. The ANT1 isoform encoded by *SLC24A4* is found at highest levels in the muscle, heart, and brain, and it is found in both dominant and recessive disease^[304]^. The dominant forms induce adult onset CPEO and mtDNA deletions, and rarely with childhood onset with severe respiratory compromise, hypertrophic cardiomyopathy, seizures, and mtDNA depletion and early death. The recessive form causes childhood/early adult onset of myopathy and cardiomyopathy^[305]^. The carrier SLC25A10 transports dicarboxylates and phosphate across the IMM. Inhibition of this function induces marked reduction in glutathione and a progressive form of epileptic encephalopathy with severe hypotonia^[306]^. There are two isoforms of AGC: one synthesized by *SLC25A12* and expressed mostly in muscle and nervous tissue, and the other isoform by *SLC25A13*. *SLC25A12* encodes the solute carrier AGC1, which catalyzes the unidirectional exchange between intra-mitochondrial asparate and cytosolic glutamate. AGC1 is a component of the malate-aspartate shuttle. Variants in *SLC25A12* are associated with severe hypotonia, arrested development, seizures, and global cerebral hypomyelination^[307,308]^. Variants in the *SLC25A13* gene encoding AGC2 induce adult onset type II citrullinemia, due to the specific loss of liver argininosuccinate synthetase^[309]^. Variants in *SLC25A15* produce a disorder of the urea cycle, hyperornithinemia-hyperammonemia-homcitrulluria, due to reduce transport of ornithine by the carrier ORC1^[310]^. SLC25A16 protein is located in the IMM and is thought to transport coenzyme A, but this has not been conclusively demonstrated. Recessive variants in *SLC25A16* are associated with severe fingernail dysplasia^[311]^. *SLC25A20* encodes the carnitine-acylcarnitine translocase that is responsible for the transport of long-chain fatty acids from the cytoplasm to the matrix for β-oxidation, via the carnitine shuttle. The translocase is embedded in the IMM and transfers acylcarnitine from carnitine palmitoyltransferase I in the OMM to carnitine palmitoyltransferase II in the IMM. Recessive variants induce an early infancy lethal condition manifested by hypoketotic hypoglycemia, cardiomyopathy, hepatopathy, and muscle weakness^[312,313]^. The protein transporter of 2-oxoadipate and 2-oxoglutarate across the IMM is SCL25A21. Biallelic variants in *SCL25A21* inhibit the degradation of tryptophan and lysine, which in turn alters the generation of NADH and acetyl-CoA, with corresponding elevations in intracellular quinolinic acid and oxopate, and together they induce a neuropathy with a spinal muscular atrophy-like disease^[314]^. In addition to SCL25A12, glutamate and associated proton (H^+^) is transported into the intermembrane space and into the matrix by SLC25A22 (GC1) and SLC28A18 (GC2). Once in the matrix, glutamate is converted into α-ketoglutarate and ammonia^[315]^. Biallelic loss of function variants in *SLC25A22* result in severe early onset malignant partial migrating seizures of infancy and early epileptic encephalopathy^[316,317]^. *SLC25A24* encodes one of the five ATP-Mg/Pi carriers. APC1 (mitochondrial ATP-Mg/Pi carrier isoform 1), whose main function is the exchange of ATP-Mg or ADP for phosphate across the IMM, is modulated by extra-mitochondrial Ca^2+[318]^. Biallelic variants induce a rare syndrome of bone dysplasia of the skull and fingers, distinctive facial dysmorphology, and prenatal and postnatal growth retardation with early demise due to decreased mitochondrial ATP synthesis^[319]^.

S-adenosylmethionine (SAM) is required for methylation of target proteins. The mitochondrial SAM carrier is encoded by *SLC25A26* and is the sole entry mechanism into mitochondria. Methylation is required for nucleic-acid modifications and ETC functioning. Variants in *SLC25A26* induce a range of phenotypes, from neonatal early death to acute episodes of cardiopulmonary failure and progressive muscle weakness^[320]^. The gene *SLC25A32* encodes the flavin adenine dinucleotide (FAD), which transports FAD into the mitochondria, needed for ETC activity and FAD-dependent cofactor requiring enzymatic activity^[321]^. Recessive variants induce a wide range of disease, from recurrent muscle exercise intolerance to early onset ataxia, myoclonia, dysarthria, and muscle weakness^[322]^. The SLC25A42 carrier is responsible for the importation of coenzyme A across the inner membrane in exchange for deoxyadenine nucleotides and ADP. Recessive variants in *SLC25A42* induce a variety of clinical features and range from isolated myopathy to movement disorders, encephalopathy, seizures, and developmental regression in other patients^[247]^. Increased iron deposition in the basal ganglia was reported in the globus pallidi and substantia nigra.

Nuclear-encoded mitochondrial sideroblastic anemias demonstrate ringed sideroblasts in erythroid precursor cells with pathologic iron deposits within mitochondria. Two mitochondrial specific genes, one X-linked, *ALAS2*, and one autosomal recessive, *SLC25A38*, represent two etiologies. *ALAS2* encodes the first enzyme in heme synthesis in the condensation of glycine and succinyl-CoA to form ALA, while the solute carrier *SLC25A38* is hypothesized to be the transporter of glycine across the IMM^[323]^. As noted above, other mitochondrial localized gene products *SLC19A2*, *PUS1*, *ABCB7*, and *GLRX5* also produce siderobastic anemia, but without intra-mitochondrion iron deposits.

The SLC25A42 carrier is responsible for the importation of coenzyme A across the inner membrane in exchange for deoxyadenine nucleotides and ADP. Recessive variants in *SLC25A42* induce a variety of clinical features and disease onset, from isolated myopathy to combinations of movement disorders, encephalopathy, seizures, and developmental regression^[247]^. Increased iron deposition in the basal ganglia was reported in the globus pallidi and substantia nigra. Unlike other members of the solute carrier family, SLC25A46 localizes to the OMM and its precise function remains unknown^[324]^. SLC25A46 has been shown to interact with both OPA1 and MFN2 suggesting a direct role in fusion/fission dynamics, but also forms a complex with the cristae remodeling protein MIC60^[325]^. Variants in *SLC25A46* express range of disease, although all patients express optic atrophy and axonal neuropathy. There are patients who also express Leigh syndrome, ponto-cerebellar hypoplasia type 1, diffuse brain and cerebellar atrophy, or ataxia^[324,325]^.

Calcium uptake enables the cytosol to communicate and regulate energy demand within the mitochondrion, resulting in activation of ATP production. Transport occurs through a specialized channel known as the uniporter located in the IMM. The uniporter complex comprises three transmembrane components, mitochondrial calcium uniporter (MCU), MCUb, and essential MCU regulator (EMRE), and two peripheral components of mitochondrial calcium uptake 1 (MICU1) and MICU2^[326]^. As calcium levels rise, the inhibitory effect of MICU1 and MICU2 is removed via a conformational change allowing calcium entry^[327]^. Recessive variants in *MICU1* produces elevations of creatine kinase with normal lactate levels and ETC activities, but induce a range of symptoms of muscle weakness, fatigue, cognitive delay, and facial dysmorphism^[328]^. Variants in the *MICU2* gene have been shown to give rise to severe cognitive impairment, spasticity, and white matter changes^[329]^.

The Zrt-Irt-like protein (ZIP) family is encoded by the *SLC39A8* gene and is responsible for metal transport, Mn^2+^, Zn^2+^, Se^4+^, and Co^2+^, across plasma membrane or intracellular organelles. Specific recessive variants in *SLC39A8* induce reduced Mn^2+^ levels in mitochondria, which produces profound developmental delay, dystonia, failure to thrive, and Leigh syndrome^[330]^.

The mitochondrial pyruvate carrier (MPC) is a protein complex consisting of two subunits, MPC1 and MPC2^[331]^. There are two recessive variants in *MPC1*; one results in loss of the MPC complex and the other with less active MPC complex. The former induces early death with respiratory, cerebral atrophy, neurological deterioration, and periventricular leukomalacia. The latter produces a milder phenotype of psychomotor retardation, hypotonia, seizures, peripheral neuropathy, and visual impairment^[332]^.

#### Pyruvate dehydrogenase complex

Pyruvate dehydrogenase complex (PDHC) lies in the matrix and catalyzes the rate-limiting step in the aerobic oxidation of pyruvate to acetyl CoA. This multimeric complex comprises copies of three enzymatic subunits, namely pyruvate dehydrogenase (E1), dihydrolipoamide transacetylase (E2), and dihydrolipoamide dehydrogenase (E3), and an E3 binding protein (BP). E1 complex consists of two alpha and two beta subunits; the gene encoding the E1α subunit, *PDHA1*, is X-linked and E1β, *PDHB* is autosomal located. Activity is regulated by reversible phosphorylation of the E1α subunit that is controlled by a family of specific PDHC kinases and phosphatases^[333]^. Recessive variants in all of the subunits; E1α (*PDH1A*), E1β (*PDHB*), E2 (*DLAT*), E3 (*DLD*), and E3BP (*PDHX*) subunits; or PDH phosphatase (*PDP1*) induce disease. Clinically, there is a wide range of findings with most patients having multiple symptoms. The most common finding is developmental delay and hypotonia, but other symptoms can be present such as seizures, microcephaly, ataxia, facial dysmorphism, optic atrophy, ptosis, involuntary movements, and spasticity^[334]^. The X-linked pyruvate dehydrogenase kinase isoenzyme (*PDK3*) gene is one of the four isoenzymes that negatively regulate the activity of PDHC by reversible phosphorylation of E1α. Variants in *PDK3* induces an X-linked Charcot-Marie-Tooth neuropathy^[335]^.

#### Krebs cycle and matrix metabolism

*Aconitase 2* (*ACO2*) encodes the mitochondrial aconitase, which converts citrate into isocitrate via a cisaconitate intermediate in the Krebs cycle. Recessive variants produce a variety of phenotypes ranging from infantile cerebellar-retinal degeneration with optic atrophy, seizures, and severe encephalopathy to cerebellar ataxia without optic atrophy and spastic paraplegia^[336]^. Isocitrate dehydrogenase is a tetramer composed of two α, one β, and one γ subunit. This enzyme converts isocitrate to α-ketoglutarate and generates NADH required at the first step of the ETC reaction. Biallelic recessive variants in the a and b genes, *IDH3A* and *IDH3B*, induce retinal degeneration and encephalopathy^[337,338]^. Fumarase hydratase converts fumaric acid to L-malate. Recessive variants induce a broad range of phenotypes ranging from severe encephalopathy to hypotonia, seizures, cortical malformations, and facial dysmorphism^[339]^. Haploinsufficiency predisposes to multiple cutaneous and uterine leiomyomatosis^[340]^. The *MDH2* product, malate dehydrogenase, converts malate into oxaloacetate. Biallelic recessive variants induce early onset hypotonia, psychomotor delay, and seizures^[341]^.

Hereditary spastic paraplegias are heterogeneous neurological disorders with pyramidal symptoms predominantly affecting the lower limbs. Autosomal dominant spastic paraplegia 9A (SPG9A) and recessive spastic paraplegia 9B (SPG9B) are induced by variants in the *ALDH18A1* gene. The protein product of *ALDH18A1* is D^1^-pyrroline-5-carboxylate synthetase (P5CS) that converts glutamate to pyrroline-5-carboxylate. The intermediate enters proline biosynthesis from glutamate metabolism into ornithine in the urea cycle^[342]^.

Nicotinamide adenine dinucleotide (NAD^+^) and nicotinamide adenine dinucleotide phosphate (NADP^+^) and their reduced forms NADH and NADPH are cofactors in oxidative-reduction reactions. NAD^+^ and NADH are involved in catabolic reactions and NADP^+^ and NADPH in anabolic reaction. The ETC is dependent on NADH for electron transport. The NAD de novo synthesis pathway begins with dietary *L*-tryptophan and is converted through a series of reactions to form NAD, the kynurenine pathway. Recessive variants in *KYNU* and *HAAO* encoding two enzymes that convert 3-hydroxykynurenine to 3-hydroxyanthranilic acid to 2-amino-3-carbvoxymuconate-6-semialdehyde have been found to induce congenital vertebral and heart malformations^[343]^. There are two NAD kinases, one found in the cytoplasm and one in the mitochondria^[344]^. The NAD kinase found exclusively in the mitochondria, NADK2 (previously noted as C5orf33), utilizes ATP or inorganic polyphosphate to form NADP^+^. NADK2 also acts as a molecular chaperone that activates and stabilizes alpha-aminoadipic semialdehyde synthase. Recessive variants in *NADK2* induce malformation of cortical development, ataxia, astatic myoclonic epilepsy, optic atrophy, and psychomotor retardation^[345,346]^. This is one of the few mitochondrial diseases that there is a treatment for: the use of a lysine restricted diet and vitamin therapy with pyridoxal phosphate induces clinical improvement with resolution of epilepsy and gait improvement^[345]^.

The double bonds of NADH and NADPH are prone to hydration, which occurs spontaneously at mildly acidic pH, at elevated temperature, or enzymatically by glyceraldehyde 3-phosphate dehydrogenase to form *R*- and *S*-epimers. When this happens, neither can act as electron donors, but make the metabolite toxic to cells. The mitochondria have a detoxification enzyme encoded by *NAXE*, which eliminates the *R*-epimer^[347]^. Rare patients with recessive variants produce a rapidly progressive neurological loss, leading to coma, global brain atrophy, and death^[347]^.

There are two fatty acid synthesis pathways in humans, the cytosolic pathway (FAS1) and the mitochondrial pathway (FAS2). The enzyme trans-2-enoyl-CoA reductase, encoded by *MECR*, catalyzes the last step in the FAS2 pathway. Recessive variants in *MECR* induce childhood onset dystonia and optic atrophy^[348]^.

The conversion of pyrophosphate to orthophosphate (Pi) occurs by a family of inorganic pyrophosphatases (PPA), found both in the cytoplasm and within the mitochondrial matrix. The needs of mitochondrial DNA, RNA, protein, and lipid synthesis rests on the activity of the mitochondrial PPA2. Biallelic *PPA2* variants display seizures, mildly delayed motor milestones, and cardiac arrhythmia^[349]^. The defining feature was sudden death due to cardiac malfunction early in life, within the classification of an event named sudden unexpected death in infancy. Most of the patients demonstrated a viral prodrome or a small amount of alcohol in older patients^[350,351]^.

ECHS1 is a key component of the β-oxidation of short- and medium-chain fatty acids, responsible for the second step of oxidation. It is also involved in the catabolism of isoleucine and valine. In valine catabolism, the two intermediates methacrylyl-CoA and acryloyl-CoA are highly reactive and can react with a variety of sulfhydryl groups containing compounds. If in excess, the random binding of sulfhydryl groups is toxic to cells. Recessive *ECHS1* variants induce leukoencephalopathy, changes in the basal ganglia consistent with Leigh syndrome, hypotonia, global developmental delay, cardiomyopathy, and early death^[352,353]^. 3-Hydroxyisobutyryl-CoA hydrolase (HIBCH) is the enzyme step, just upstream of ECHS1 activity in valine metabolism. Biallelic variants in *HIBCH* manifest as neurodevelopmental delay, dystonia, and ataxia with MRI findings consistent with Leigh syndrome^[354]^.

L-2 hydroxyglutarate dehydrogenase is a FAD-dependent enzyme that catalyzes the conversion of L-2-hydroxygluratic acid to 2-ketoglutarate. L-2-hydroxyglutaric acid elevation in urine, cerebrospinal fluid, and plasma is pathognomonic for *L2HGDH*-induced disease^[355]^. Patients with biallelic *L2HGDH* variants have exclusive neurological findings of psychomotor retardation, cerebellar ataxia, macrocephaly, and epilepsy^[356]^. Recessive and rare dominant *D2HGDH* variants in the chiral configuration enzyme, D-2-hydroxyglutarate dehydrogenase induce D-2-hydroglutaric acid to 2-ketoglutarate, and patients present with epilepsy, hypotonia, and psychomotor retardation^[357]^.

The OXPHOS generating ATP system presents a problem to the cell: the reduction of oxygen produces superoxide, which is dismutated to hydrogen peroxide (H_2_O_2_). Tight regulation of H_2_O_2_ is critical for physiological cell signaling and avoiding non-specific oxidative damage. The interlinkage of thioredoxin and glutathione prevent excessive H_2_O_2_ under normal conditions. The thioredoxin system is composed of thioredoxin 2 (TXN2), thioredoxin 3 (PRDX3), and peroxiredoxin 5 (PRDX5), and it is responsible for removing excess H_2_O_2_^[358]^. Recessive variants in *TXN2* have demonstrated microcephaly, cerebral atrophy, psychomotor delay, epilepsy, optic atrophy, and retinopathy^[359]^.

The final pathway of sulfur containing amino acids, methionine, and cysteine occurs in the mitochondrial matrix with the degradation of hydrogen sulfide (H_2_S). H_2_S is also produced by gut anaerobic bacteria and metabolized in the matrix. Ethylmalonic encephalopathy protein 1 (ETHE1) is an iron containing sulfur dioxygenase in the matrix pathway. Biallelic variants in *ETHE1* demonstrate early onset progressive psychomotor retardation, cerebral hemorrhagic lesions, chronic diarrhea, and early death^[360]^. The exact mechanism of mitochondrial dysfunction remains unclear, but cell culture studies suggest impairments of ATP production, dynamics, and disruption of ER-mitochondria contacts^[361]^.

## CONCLUSIONS AND FUTURE DIRECTIONS

Mitochondrial disorders are heterogeneous in clinical presentation and genetic etiology. Individual disorders are rare, but as a group they represent a common inborn error of metabolism. Phenotypically, a range of organ involvement is the signature of the disease with those organs requiring the most energy expressing a range of clinical features. However, tremendous overlap exists in the clinical features at presentation, age of presentation, and progression of disease. Since there are no pathognomonic tests or findings identifying a patient with mitochondrial disease, diagnosis is problematic. Previously, biochemistry of bodily fluids, structural (muscle biopsy), neuroimaging, and clinical examination with thorough history were required for diagnosis. In the initial thirty years after the first description, the expansion of disease steadily grew, albeit slowly. To this day, the classic syndromes remain steadfast in phenotype [Table 1], but the range of clinical presentation suggests a wider complement of genetic etiologies. The rise of commercially available massive parallel sequencing platforms has transformed the diagnosis and demonstrated an expansive and diverse group of inherited diseases [Tables 3 and 4]. The increasing range of genetic variants demonstrating mitochondrial dysfunction is pushing the boundaries of the older nosology of diseases, genes involved directly in OXPHOS function, and those modulating other mitochondrial physiologies. There is a trend towards a physiologically based genetic classification. I have tried to use such a scheme in writing this review.

The lack of a precise definition of primary mitochondrial disease has created diagnostic dilemmas in both patient identification and inappropriate phenotypes being classified as mitochondrial disease based on biochemical and/or muscle findings^[362]^. Isolated genetic findings have shown that unvalidated variants once thought to give rise to primary mitochondrial disease are found in healthy individuals^[29]^. Others have thought that primary disease is based solely on genetic variants encoding OXPHOS proteins directly or affecting OXPHOS function by impacting production of the complex machinery needed to run the OXHOS process^[363]^. However, the complete ascertainment of mitochondrial physiological functioning is unknown at present. The advent of widespread genetic testing and validation testing will solidify primary mitochondrial disease diagnosis and, in time, expand those non-primary diseases that have overlapping phenotypes and mitochondrial insults. The understanding of these secondary or environmental mitochondrial insults will help providers to forge better target therapies for inflammation, myopathy, diabetes, neurodegeneration, and ecogenetic variants altering disease^[364]^.

The development of effective treatments for mitochondrial diseases represents an enormous challenge. The extensive range of altered mitochondrial physiology makes clinical management of affected individuals challenging. In fact, the most recent Cochrane review found there are no reliable and reproducible treatments for mitochondrial diseases^[365]^. The advent of expanded genetic testing has identified genetic abnormalities with altered cofactor availability, which can be amenable to vitamin and/or cofactor supplementation for disease treatment^[366]^. Although small in number, these disorders should not be missed as they represent treatable conditions.

Genetic treatment of disease has recently entered the clinical realm as the Federal Drug Agency (FDA) approved the use of a single dose of intravenous adeno-associated virus serotype 9 carrying human survival motor neuron gene (*SMN*) complementary DNA for patients with spinal motor atrophy^[367]^. However, genetic treatment is currently very limited in scope, only used in several diseases, and has not been FDA approved in mitochondrial disease. Without available treatments, genetic diseases have relied on counseling, prenatal testing, preimplantation genetic diagnosis, and using surrogate donor or adoption for family decision making. However, in nuclear diseases, the rare occurrence makes these options only available once a previous sibling has been identified or strong family history. The unique inheritance of mtDNA variants has introduced a possible alternative to predictive prenatal counseling, but it relies on precise genetic testing and biological validation. All mtDNA passed on into oocytes occurs after conception within the woman and is sorted by a process named bottleneck^[53]^. This creates random amounts of mutated mtDNA in different oocytes, and therefore the result of any pregnancy is uncertain. Prenatal testing is only viable for those women with low risk of mtDNA transmission and who would consider termination. In 2015, the House of Lords in the United Kingdom enabled mitochondrial replacement^[368]^. Mitochondrial replacement or donation involves the removal of the nuclear DNA from a mother with pathological mtDNA into an oocyte (metaphase II spindle transfer) or zygote (pronuclear transfer) to a donor woman’s oocyte with normal mtDNA^[368]^. Currently, this process is only legally supported in the United Kingdom, whereas, in the rest of the world, it is either not allowed or there is a lack of legal regulation. The long-term safety of this technique remains uncertain and approval for use is still awaiting confirmation.

Other than the few disorders that can be treated by cofactor/vitamins, the vast majority of mitochondrial diseases remain without satisfactory treatments. An example of the advancement in mitochondrial medicine has been the proliferation of clinical trials^[369]^. Unfortunately, early results of some of these trials has still not led to FDA approved medications for mitochondrial disease. However, the breadth of cellular metabolic integration under mitochondrion control has been greatly expanded by gene discovery. The expansive range of mitochondrial physiological functions suggests no single treatment will correct all disease. Therapy will have to be targeted to specific mechanisms. Increased gene discovery with biological integration of multiple physiological functions will need to drive precision drug discovery for various mitochondrial diseases.

## Figures and Tables

**Table 1.** Classical mitochondrial syndromes due to mtDNA- and nuclear-encoded variants

Clinical syndrome	Clinical phenotypes	mtDNA/nuclear-encoded	Age of onset
Pearson	Exocrine pancreatic dysfunction,sideroblastic anemia	mtDNA	Infancy
Kearns-Sayre	Ophthalmoplegia, RP, cardiac Conduction block, diabetes, short Stature, myopathy	mtDNA	Childhood
CPEO	Ophthalmoplegia, ptosis, myopathy	mtDNA/Nuclear	Adult
LHON	Optic atrophy	mtDNA	Adolescence/adult
Leigh	Psychomotor delay, dystonia, seizures	mtDNA/Nuclear	Childhood
NARP	RP, peripheral neuropathy, ataxia,	mtDNA	Adolescence/adult
MELAS	Metabolic strokes, seizures, migraine Blindness, dystonia, myopathy, short Stature	mtDNA	Adolescence/adult
MIDD	Diabetes, sensorineuroal hearing loss	mtDNA	Adolescent/adult
MERRF	Myoclonus, myoclonic seizures, Myopathy, sensorineural hearing loss Lipomatosis	mtDNA	Adolescent/adult
AHS	Seizures, hepatopathy, psychomotor delay, GI dysmotility, peripheral neuropathy, blindness	Nuclear	Childhood
Barth	Dilated cardiomyopathy, cyclic neutropenia, myopathy	Nuclear	Childhood
MNGIE	Leukoencephalopathy, GI dysmotility, Ophthalmoplegia, Cachexia, peripheral neuropathy	Nuclear	Adult
Friedreich Ataxia	Progressive spinocerebellar ataxia dysarthria, muscle weakness, diabetes cardiomyopathy	Nuclear	Adolescent/adult

CPEO: chronic progressive external ophthalmoplegia; RP: retinitis pigmentosa; LHON: Leber hereditary optic neuropathy; MELAS: mitochondrial encephalomyopathy, lactic acidosis and stoke-like episodes; MIDD: maternal-inherited diabetes and sensorineural hearing loss; NARP: neuropathy, ataxia, and retinitis pigmentosa; MERFF: myoclonus, epilepsy with ragged red fibers (also named myoclonic epilepsy with red ragged fibers); AHS: Alpers Huttenlocher syndrome; GI: gastrointestinal tract; MNGIE: mitochondrial neurogastrointestinal encephalomyopathy

**Table 2.** Clinical Features of Mitochondrial Disease

Organ system	Clinical feature
Brain	Encephalopathy, microcephaly, ataxia, seizures, dementia, stroke, Parkinsonism, developmental delay and regression, intellectual impairment, psychiatric disorder, autism, cerebellar hypotonia, dystonia
Peripheral Nerve	Sensory and axonal neuropathy, dysautonomia, aberrant temperature regulation, orthostatic hypotension, abnormal sweating
Special Senses	Sensorineural hearing loss, optic atrophy, retinitis pigmentosa, cataract, aminoglycoside hearing loss
Muscle	Ophthalmoplegia, eyelid ptosis, myopathy, muscle cramping, exercise intolerance, hypotonia
Respiratory	Respiratory failure
Cardiac	Cardiac conduction defect, cardiomyopathy (dilated, restrictive, hypertrophic)
Renal	Proximal renal tubular dysfunction (Falconi syndrome), nephrotic syndrome, Barrter syndrome, tubulointerstitial disease
Endocrine	Diabetes mellitus, hypogonadism, hypoparathyroidism, infertility, short stature (not growth hormone related), growth hormone deficiency, adrenal insufficiency, exocrine pancreatitis
Gastrointestinal	Dysphagia, cyclic vomiting, pseudo-obstruction, gastrointestinal dysmotility
Hepatic	Hepatopathy, nonalcoholic steatohepatitis
Hematological	Siderblastic and macrocytic anemia, pancytopenia, erythrocyte failure
Dermatological	Lipomatosis
Skeletal	Kyphosclerosis, bone marrow failure

**Table 3.** Genes directly related to oxidative phosphorylation biogenesis that have been linked to disease.

OXPHOS ETC subunits	
Complex I	
Mitochondrial-encoded subunits:	*Mt-ND1, Mt-ND2, Mt-ND3, Mt-ND4, M-ND4L, Mt-ND5,Mt-ND6*
Nuclear-encoded subunits:	*NDUFA1, NDUFA2, NDUFA9, NDUFA10, NDUFA11, NDUFA12, NDUFA13, NDUFB3, NDJFB9, NDUFB10, NDUFB11, NDUFS1, NDUFS2, NDUFS3, NDUFS4, NDUFS6, NDUFS7, NDUFS8, NDUFV1, NDUFV2*
Complex II	
Nuclear-encoded subunits:	*SDHA, SDHB, SDHD*
Complex III	
Mitochondrial-encoded subunits:	*Mt-CYB*
Nuclear-encoded subunits:	*CYC1 UBCRB, UQCRC2*
Complex IV	
Mitochondrial-encoded subunits:	*Mt-CO1, Mt-CO2, Mt-CO3*
Nuclear-encoded subunits:	*COX41, COX412, NDUFA4*
Complex V	
Mitochondrial-encoded subunits:	*Mt-ATP6, Mt-ATP8*
Nuclear-encoded subunits:	*ATP5A1, ATP5E*
OXPHOS assembly factors	
Complex I	
Nuclear-encoded genes:	*ACAD9, FOXRED1, NDUFAF1, NDUFAF2, NDUFAF3, NDUFAF4, NDUFAF5, NDUFAF6, NUBPL, TIMMDC1, TIMEM126B*
Complex II	
Nuclear-encoded genes:	*SCHAF1*
Complex III	
Nuclear-encoded genes:	*BCSIL, LYRM7, TTC19, UQCC2*
Complex IV	
Nuclear-encoded genes:	*COA3, COA5 COA6, COA7, COXIO, COX14, SC01, SCO2, COX15, COX20, PET100, APOFT1, SURF1, PET 117*
Complex V	
Nuclear-encoded genes:	*ATPAF2, TMEM70, USMG5*
Replication/homeostasis, transcription, translation	
mtDNA replication/homeostasis	
Nuclear-encoded genes:	*POLG, POLG2, DNA2, MGME1, RNASEH1 TFAM, TWNK*
Nucleotide pools	
Nuclear-encoded genes:	*ABAT DGUOK MPV17, RRM2B, SAMHD1, SUCLA2, SUCLG1, TK2, TYMP*
Electron carriers	
Nuclear-encoded genes:	*COQ2, COQ4, COQ5, COQ6, COQ7, COQ8A, COQ9, PDSS1, PDSS2, CYCS, HCCS*
Mt-tRNA biogenesis	
Mitochondrial-encoded genes:	*MT-TA, MT-TC, MT-TD, MT-TE, MT-TF, MT-TG, MT-TH, MT-Ti, MT-TK, MT-TL1, MT-TL2, MT-TM, MT-TN, MT-TP, MT-TQ, MT-TR, MT-TS1, MT-TS2, MT-TT, MT-TV, MT-TW, MT-TY,*
Nuclear-encoded genes:	*GTPP3, MTFMT, NSUN3, PUS1, ORSL1, TRIT1, TRMT5, TRMU, TRNT1, MTO1*
Mt-tRNA aminoacylation	
Nuclear-encoded genes:	*AARS2, CARS2, DARS2, FARS2, GARS, HARS2, IARS2, KARS2, LARS2, MARS2, NARS2, PARS2, RARS2, SARS2, TARS2, VARS2, WARS2, YARS2*
mtRNA expression/processing	
Nuclear-encoded genes:	*ELAC2, FASTKD2, HSD17810, LRPPRC, MRM2, MTPAP, PNPT1, TRMG10C*
Mitochondrial ribosome biosynthesis	
Mitochondrial-encoded genes:	*mt-RNR1*
Nuclear-encoded genes:	*MRPL12, MRPL44, MRP57, MRPS16, MRPS22, MRPS23, MRPS34, ERAL1, MMPL3*
Translation	
Nuclear-encoded genes:	*C12orf65, GRM1, GFM2, RMD1, TSFM, TUFM, TAC*

**Table 4.** Nuclear-encoded genes involved in mitochondrial physiology not directly related to oxidative phosphorylation that induce disease

Fe-S cluster biosynthesis:	*ABCB7, BOLA3, FDX1L, FDXR, FXN, GLRX5, IBA57, ISCA2, ISCU, LYRM4, NFS1, NFU1, IREB2, C19orf12*
Enzyme co-factors:	*COASY, FLAD1, LIAS, LIPT1, PANK2*
Protein quality control:	*AFG3L2, CLPP, LONP1, SPG7, YME1L1, PARL, PMPCB, IMMP2L, HTRA2, XPNPEP3*
Lipid modification:	*AAD3A, CHKB, PLA2G6, SERAC1, TAZ*
Protein Import/processing:	*AKG, AIFM1, DNAJC19, GFER, M/PEP, PMPCA, TIMM8A, T/MM50, AMT, GLDC*
Mitochondrial morphology:	*C19orf70, DNM1L, GDAP1, MFF, MFN2, MSTO1, OPA1, SA CS, SLC25A46, STA T2, TRANK1, VPS13D, VPS13A*
Matrix metabolism:	*D2HGDH, ECHS1, ETHE1, H/BCH, L2HGDH, NAKE, TXN2*
Metabolic transport:	*SLC19A1, SLC25A1, SLC25A3, SLC25A4, SLC25A10, SLC25A12, SLC25A13, SLC25A15, SLC25A16, SLC25A19, SLC25A20, SLC25A21, SLC25A22, SLC25A24, SLC25A26, SLC25A32, SLC25A38, SLC25A42, SLC25A46, SLC2A13, MICU1, MICU2, MPC1*
TCA cycle and metabolism:	*ACO2, ALDH18A1, DLA T DLD, FH, HAAO, IDH3A, IDH3B, KYNU, MDH2, MECR, NADK2, PDHA1, PDHB, PDHX, PDK3, PDP1, PPA2*
Autoptosis/autophagy:	*HTRA2, VPS13C*
Unclear function:	*APOPT1, C19orf!2, C1QBP, FBXL4, OPA3, RTN4IP1, SFXN4, TMEM65, CYP2U1*
